# One direction? Cultural aspects of the mental number line beyond reading direction

**DOI:** 10.1007/s00426-024-02038-4

**Published:** 2024-12-23

**Authors:** Merve Bulut, Lilly Roth, Narjes Bahreini, Krzysztof Cipora, Ulf Dietrich Reips, Hans-Christoph Nuerk

**Affiliations:** 1 Department of Psychology, Yaşar University, Izmir, Turkey; 2 Department of Psychology, University of Tuebingen, Schleichstrasse 4, 72072 Tuebingen, Germany; 3 Department of Mathematics Education, Loughborough University, Loughborough, UK; 4 Department of Psychology, University of Konstanz, Constance, Germany

## Abstract

*Spatial-Numerical Associations* (SNAs) refer to the demonstrations of spatial processing of numbers. The *Mental Number Line* (MNL) is a representation model describing numbers as aligning left-to-right (LR) and was suggested to account for directional biases in participants’ responses during numerical tasks. One common behavioral demonstration of this is the *Spatial-Numerical Associations of Response Codes* (SNARC) effect, which describes faster left-/right-hand responses to smaller/larger numbers, respectively. The MNL, and, consequently, directional SNAs, show variabilities across different cultures. Reading direction is considered to be the main factor in explaining these differences. In line with this, individuals with right-to-left (RL) reading habits show a weaker or even reverse SNARC effect. In the present study, we investigated whether SNAs are influenced not only by reading direction, but also by cultural directional preferences such as drawing lines, arranging objects, imagining objects (i.e., rightward or leftward facing), or representing events in time (i.e., mentally representing the past/future on the left/right, respectively). To test this hypothesis, we measured the cultural directional preferences and the SNARC effect across three cultures in an online setup; German, Turkish, and Iranian. LR preferences in the Cultural Directional Preferences Questionnaire were most prominent in German participants, intermediate in Turkish participants, and least prominent in Iranian participants. In line with this, the LR SNARC effect was strongest in German, intermediate in Turkish, and weakest (but not RL) in Iranian culture. These findings suggest that cultural directional preferences are involved in the emergence of adult SNAs in addition to the reading direction.

**Supplementary Information:**

The online version contains supplementary material available at 10.1007/s00426-024-02038-4.

## Introduction

Numeracy is a crucial skill for understanding the external world and has many applications in everyday life. Therefore, it is essential to understand how humans process numbers and create a meaningful mental representation of them. One core concept related to elementary number processing is the strong cognitive link between numerical magnitude and space. *Spatial-Numerical Associations* (SNAs) are a group of behavioral and neural demonstrations indicating that numerical information is spatially represented (for a review see Cipora et al., 2018).

One of the most reported behavioral demonstrations of SNAs is the *Spatial-Numerical Association of Response Codes* (SNARC; Dehaene et al., 1993). In a given numerical range, smaller numbers are responded to faster with left-side buttons and larger numbers with right-side buttons. The SNARC effect is thought to be a result of the left-to-right (LR) orientation of the *Mental Number Line* (MNL), on which smaller numbers are mapped on the left and larger ones on the right (Dehaene et al., 1993; for alternative accounts see Gevers et al., 2010; van Dijck & Fias, 2011; Schroeder et al., 2017; Cipora et al., 2020).

One line of research focuses on whether this close link between space and number has a biological basis or is culturally acquired. Animal studies suggest that LR spatial processing of magnitudes is innate by demonstrating LR processing of magnitudes in newly born chicks (Rugani et al., 2015) and macaque monkeys (Adachi, 2014; Drucker & Brannon, 2014). This view is also supported by evidence from studies with preliterate infants. Specifically, infants seem to process increasing magnitudes in LR fashion (e.g., de Hevia et al., 2014; Bulf et al., 2016, Di Giorgio et al., 2019; for a review see McCrink & Opfer, 2014). These studies show that the spatial processing of magnitudes appears very early in development. However, since these studies only include Western infants born and raised in the LR-oriented cultural environment, their behaviors may be highly influenced by their noticing of environmental clues (see McCrink & de Hevia, 2018; Patro et al., 2016a for discussions). Importantly, —whether or not these tendencies are innately present—it is well-demonstrated that culture can shape SNAs, as we will see in the following section. The present paper focuses on how cultural directionalities beyond reading/writing direction (referred to as reading direction in the text hereafter) can influence the spatial processing of numbers.

## The influence of reading direction on SNAs

Reading direction was proposed as the source of SNAs soon after the SNARC effect was first observed. Already in the first systematic investigation of the SNARC effect, Dehaene et al. (1993; Exp.7) reported that it was absent among Iranian participants who live in France. The right-to-left (RL) reading direction of the Farsi (also known as Persian) language was suggested as an explanation. The results were similar when Arabic and Eastern Arabic (typically used in Farsi) numbers were used as stimuli. Dehaene et al. concluded that the absence of the SNARC effect in Farsi speakers could be related to the fact they were using both LR (French) and RL (Farsi) reading directions. An additional analysis revealed a significant relationship between the time participants spent in France and the SNARC effect; the longer they spent in France, the stronger the SNARC effect.

Shaki et al., (2009) examined the effect of reading direction on the SNARC effect in a cross-cultural setting more systematically. On top of reading directions for text, they considered whether numbers in a given language are written LR or RL. These directions do not match in some languages, including Farsi and Hebrew (text is written from right to left, but numbers are written from left to right). They recruited three samples: Canadians (LR of both words and Arabic numbers), Israelis (RL reading of words and LR reading of Arabic numbers), and Palestinians (RL reading of both words and Eastern Arabic numbers). Canadians revealed a regular SNARC effect, Palestinians a reverse SNARC effect (i.e., faster left-/right responses to large/small numbers, respectively), and Israelis no effect. Shaki et al. (2009) also attributed the differences in the SNARC effect to reading habits (see also Zebian, 2005). The null effect in Hebrew speakers was attributed to the inconsistent reading direction of text (RL) and numbers (LR). However, one must keep in mind that in these early studies, the statistical power was poor for detecting small effect sizes due to small samples (between 11 and 20 participants, the minimum detectable effect size would have been *d* = 0.94 to *d* = 0.66 for 0.80 power, respectively) and poor measurement precision resulting from insufficient numbers of repetitions per combination of number and response-to-key assignment (between 8 and 16 repetitions), whereas at least 20 repetitions are recommended (see Cipora & Wood, 2017). At the same time, several subsequent studies demonstrated a regular SNARC effect at the group level in RL readers (Feldman et al., 2019; Hochman et al., 2024; Shaki & Gevers, 2011; Zohar-Shai et al., 2017; Supplementary Material of Cipora et al., 2019a).

The connection between reading direction on the SNARC effect is further supported by studies testing bilingual individuals with both LR (Russian) and RL (Hebrew) reading habits. The SNARC effect can alter even within a very short time after reading a Russian (regular SNARC) or Hebrew (no SNARC) paragraph (Shaki & Fischer, 2024) or even a single Russian or Hebrew number word (Fischer et al., 2009). This suggests that reading direction (and associated cultural influences) not only has long-term memory-related but also short-term priming-like influences on the SNARC effect. At the same time, the conclusion that reading manipulation can swiftly alter the SNARC has not always been supported. For instance, Pitt and Casasanto (2020) examined the causal influence of short-time reading activity on the SNARC effect by giving English-speaking participants a text in their native language either with a standard or mirror-reversed orthography. The SNARC effects after reading in a standard and reverse fashion were similar, showing no evidence of the influence of reading direction on the SNARC effect.

To sum up, while the impact of the reading direction of the SNARC is still far from fully resolved, studies looking into this effect have provided some hints about the role of other directional habits in shaping the SNARC effect. At the same time, the direction in which numbers are written is more nuanced and does not fully allow testing this hypothesis. It is important to differentiate writing numbers in a text line and the numeration systems. Although the Eastern Arabic numbers used by Palestinian Arabic speakers and Farsi speakers are written RL in line organization (i.e., one writes digits from one to five starting from right), they are written LR in the numeration system (i.e., multi-digit numbers have the highest power to ten on the left and the unit on the right as in Western languages including English; see Lopiccolo & Chang, 2021; Rashidi-Ranjbar et al., 2014). Hence, differences between Hebrew and Arabic do not include the direction of their numeration systems (i.e., they are both LR).

## The influence of cultural directional preferences on SNAs

Although the evidence reviewed above suggests that reading direction influences SNAs, it does not indicate that reading direction is the only cultural factor involved in their emergence. Patro et al. (2016b) proposed an explanation of how engaging in actions that include a spatial trajectory (such as reading a text, or horizontal ordering of items) can influence numerical magnitude processing. Accordingly, the direction of action corresponds with the time passing, spatial distance from the starting point, and the amount of objects involved. For instance, when observing the activity of arranging books on a shelf in an LR fashion, one can realize that as the action moves towards the right, more time passes, the space covered in the shelf increases, and more books accumulate. Therefore, engaging in spatially oriented behaviors or observing them may enhance the spatial processing of magnitudes.

In line with this, evidence from preschool children studies shows that SNAs are established before reading skills emerge. Hoffmann et al. (2013) found a significant regular SNARC effect with 5.5-year-old children in a color classification task of Arabic numbers (but only when a magnitude classification task of Arabic numbers had been previously performed). Moreover, with a non-symbolic magnitude comparison task, Ebersbach et al. (2014) showed that SNAs are prevalent before formal education (see also Patro & Haman, 2012). Similarly, Shaki et al. (2012) reported culture-specific object-counting preferences among preschool children consistent with the prominent reading direction (LR in British, RL in Palestinian, and no dominant preferences in Israeli preschoolers; see also Patro et al., 2015).

To explain how SNAs might be constructed by the cultural environment in preschool children, Patro et al. (2016b) proposed the *implicit instruction account*. The account suggests that SNAs in preliterate children are not necessarily innate, but can emerge from implicit learning of culture-specific directional preferences acquired by observing and interacting with parents and their social environment. To support their hypothesis, Patro et al. (2016b) trained 3- to 4-year-old preliterate children with a non-numerical task that involves spatial finger movements (LR or RL) on a touch screen to move a frog across a pond. After the training, the LR group showed a regular SNARC effect and the RL group showed a reversed SNARC effect in a numerical comparison task. These findings fit well with Patro et al. (2016a, 2016b)’s explanation of the emergence of SNAs mentioned above. Visuomotor training in a specific direction (LR or RL) enhances the spatial trajectory of longer duration, larger numerosity, and larger distance, compatible with the training direction (see also McCrink & de Hevia, 2018). Therefore, these findings suggest that directional visio-motor behaviors can establish (at least temporarily) SNAs. Göbel et al. (2018) demonstrated a similar effect of the observed reading on the counting preferences. 3- to 5-year-old English- and Arabic-speaking preliterate children changed their counting preference in line with the direction of the observed storybook reading (LR or RL). Shaki and Fischer (2023) provided further evidence for the link between counting preference and attentional SNARC in adults.

In a similar vein, Nuerk et al. (2015) proposed several mechanisms to explain how certain behaviors can influence the emergence of SNAs among preliterate children. The emergence of SNAs may result not only from observing adult reading behavior and pretending to read/write, but also from “dominant attentional-directional preferences in a society, not directly related to reading direction” (Nuerk et al., 2015, p. 3) such as drawing lines, arranging items, imagining objects (i.e., rightward or leftward facing), or representing events in time (i.e., mentally representing the past and the future on the left or right; see also Patro et al., 2016a). Here, we refer to these behaviors as cultural directional preferences, and for clarity, we separate cultural directionalities as (i) reading direction (which is obligatory in society) and (ii) cultural directional preferences unrelated to reading direction. Previous studies investigating culture's influence on adult SNAs have focused on reading direction, overlooking cultural directional preferences, even though, as summarized above, studies with preliterate children suggest that directional actions other than reading are also involved.

The postulate that factors other than reading direction are also responsible for the SNARC effect is considered in the recent *CORrelations in Experience (CORE) principle* (Pitt & Casasanto, 2020), stating that different experiences such as reading and finger counting should selectively influence different abstract representations such as MNL. Based on this, reading activity highly correlates with time and space (more time passes by when the reader moves in the text in LR or RL) but not with number and space unless the text includes numbers; therefore, there is no need to expect that there will be a causal relationship between text and MNL directions.

To summarize, cultural directional preferences can influence SNAs. This evidence comes mainly from studies with preliterate children, where cultural directional preferences seem to impact SNAs even before reading skills are acquired. In the following, we will outline the evidence pertaining to whether cultural directional preferences could be also influential in adult SNAs.

## Reading direction versus cultural directional preferences

The theoretical accounts proposed to emphasize the influence of cultural directional preferences were formulated to explain the SNAs that emerge in preliterate children in the process of development. One interesting question is whether cultural directional preferences still influence SNAs in adults. One possibility is that the effects observed among preschool children result from a transient phase in which children use spatial cues in their social environment to process magnitudes. The most common and consistent cultural directionality is reading, and therefore, reading direction would be the prominent factor determining SNAs in adulthood. However, there is another possibility, that as well as reading direction, cultural directional preferences also have an influence.

It is difficult to test whether cultural directional preferences are also involved in the emergence of SNAs in adulthood because these are frequently consistent with the reading direction of that culture (Chokron & de Agostini, 1995; Faghihi et al., 2019; Maass & Russo, 2003; Rinaldi et al., 2014; Schild et al., 2022 Vaid, 1995 for a review see Faghihi & Vaid, 2023). At first sight, it seems unlikely that there are cultures and individuals who might have opposite cultural directional preferences and reading directions. However, Turkish culture may provide this remarkable opportunity to dissociate long-term cultural directional preferences from current reading habits. In the following paragraph, we detail how this unique condition might have emerged.

The official alphabet of the Turkish language was a combination of Arabic-Farsi written RL until 1928 for around six hundred years (during the Ottoman Empire). In 1923, the modern Turkish Republic was founded and there were reforms in several areas. One of these revolutions involved replacing the Arabic-Farsi alphabet with the Latin alphabet in 1928 (Yılmaz, 2011). Even though modern Turkish and the old Ottoman Turkish languages are almost identical, after the reform, the Turkish language was written with the Latin alphabet in an LR fashion. We suggest that this switch from RL to LR direction in the script may create inconsistencies between the reading habits and cultural directional preferences in Turkish culture, because directional preferences do not change automatically with script change (see e.g., Nuerk et al., 2015, for children). It is possible that they survived to the present due to longstanding traditions of Middle Eastern culture, even apart from the previous RL reading direction. For instance, the Islamic religion, still very prominent in Turkey, recommends initiating actions from the right side. Other cultural preferences such as counting, drawing preferences of objects, and ordering preferences might also have survived, despite the change in reading directions. We investigated this issue by using a questionnaire designed to measure cultural directional preferences.

If there are mixed cultural directionalities in Turkish culture (i.e., LR reading and RL directional preferences), then we would expect weaker LR SNAs compared to a typical LR culture in which cultural directional preferences covary with the reading direction. Confirming this expectation, Bulut et al. (2023) reported no significant SNARC effect in a Turkish sample.

## The present study

In the present study, we aimed to test the *cultural directionality hypothesis:* We suggest that SNAs are influenced by the cultural environment, including reading habits and many other cultural directional preferences in daily life. These two factors are usually consistent, and thus, indistinguishable, but this might not be the case in Turkish culture due to the script direction change. Some cultural directional preferences might still be RL. Consequently, the possible inconsistencies of directionalities might weaken the LR SNAs in Turkish culture.

**Fig. 1:**
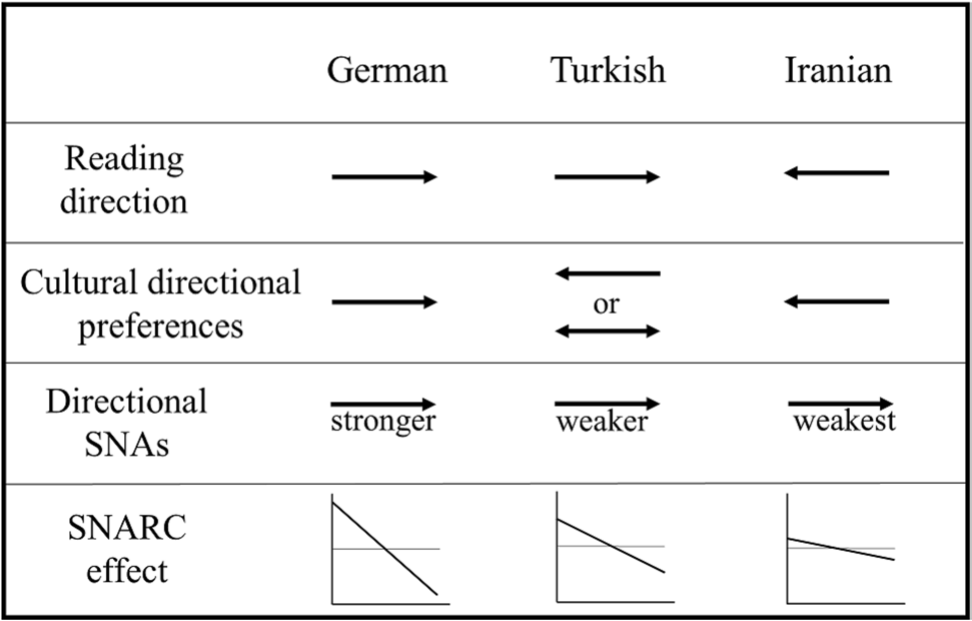
The depiction of the cultural directionality hypothesis which predicts that both reading direction and cultural directional preferences are involved in the emergence of SNAs. Turkish culture is expected to be mixed regarding reading direction and cultural directional preferences. Therefore, we expected SNAs (specifically the SNARC effect) to be less prominent than a consistent LR culture (i.e., German). Also, we expected the SNARC effect to be more prominent in Turkish than a consistent RL culture (i.e., Iranian) based on the reading direction influence. A steeper negative slope represents a stronger SNARC effect

To test the cultural directionality hypothesis, we designed a cross-cultural study comparing the effect of reading direction and cultural directional preferences on SNAs. In the existing cross-cultural SNA studies, these two elements are confounded. To disentangle cultural directional preferences from reading direction, we recruited samples from three distinct cultures. We compared directional SNAs in a Turkish sample (hypothetically mixed culture, in which the reading direction currently is LR but cultural directional preferences mostly RL) with a German (predominantly LR) and an Iranian (predominantly RL) sample. The aim of the comparison of the German and Turkish samples was to reveal any influences of cultural directional preferences since reading direction is LR in both cultures. Furthermore, comparing the Iranian and Turkish samples would reveal the influence of reading direction by reducing the involvement of cultural directional preferences, since we expect these to be similarly RL in both (see Fig. 1).

To test the cultural directionality hypothesis, we also aimed to check whether cultural directional preferences in the Turkish sample are, as hypothesized, RL or mixed, with a newly designed Cultural Directional Preferences Questionnaire (CDPQ), introduced below. We expected cultural directional preferences in Turkish participants to be more RL compared to a predominantly LR culture (i.e., German) and more LR compared to a predominantly RL culture (i.e., Iranian).

Importantly, “predominantly LR/RL cultures" does not imply that every individual or every behavior of individuals in these samples is in line with certain directions consistently. On the contrary, there is a wealth of evidence for variance in various forms of directional SNAs within cultures. For instance, only 35–45% of participants in Western samples show a reliable regular SNARC effect and 5–10% even show a reliable reverse SNARC effect (Cipora et al., 2019a, Wood et al., 2008; see also Table 3 for the prevalence of SNARC across cultures in the present study). Moreover, the SNARC effect seems to vary within participants (Roth et al., 2024b). Similarly, explicit spatial-numerical activities such as finger counting vary between individuals. Even in predominantly LR cultures (reading direction and general spatial-directional activities beyond reading), a considerable proportion of participants start finger counting with their right hand (see Hohol et al., 2018 for an overview). To conclude, said effects are typically observed at the group level. We expect Iranians to be more consistent in their RL preferences and Germans in their LR preferences compared to other samples. Based on our cultural directionality hypothesis, Turkish culture includes reverse horizontal directionalities (LR and RL) more than typical LR and typical RL cultures, which could eventually be an explanation for the weaker average SNARC effect in the Turkish sample.

### Cultural directional preferences questionnaire (CDPQ)

Directional preferences in different cultures have been widely investigated. The following preferences have been demonstrated to be consistent with reading direction (see Nuerk et al., 2015, for a review): drawing a line LR or RL (Lieblich et al., 1975; Rinaldi et al., 2014), imagining/drawing objects rightward (in RL cultures) or leftward (in LR cultures) (Vaid, 1995), aligning a timeline such that the past is on one side and the future is on the other (Tversky et al., 1991), and preferences regarding the direction/side of action/placement of the agent (Maass & Russo, 2003). Even though previous studies examined all these preferences in isolation, cultural directional preferences have yet to be combined to an assessment. For the present study, we have developed the CDPQ in which we assess horizontal directional preferences across cultures by asking the preferences of participants on certain directionalities. In this way, we could support our initial assumption that the cultural directional preferences in the Turkish sample tend towards RL more compared to the German sample and towards LR more compared to the Iranian sample. In this study, we limit ourselves to horizontal preferences as in the cultures we look at they may directly interfere with the reading direction. Even though other vertical and sagittal cultural directionalities and SNAs have been identified (see Hung et al., 2008; Ito & Hatta, 2004; Winter et al., 2015), they are orthogonal to reading direction in cultures we investigate.

### SNARC effect

To examine directional SNAs across three cultures, we used the SNARC effect which is the most widely reported directional SNA. Typically, the SNARC effect is investigated with parity judgment (PJ) and magnitude classification (MC) tasks (see Wood et al., 2008). Although the SNARC effect is observed in both task setups, it is important to differentiate them. Mainly, in PJ, participants are asked to classify numbers as odd or even and, in MC, as small or large. Magnitude processing is a part of the task instruction in MC but is task-irrelevant in PJ. Consequently, the SNARC effect observed in PJ is considered evidence for automatic magnitude processing (see Roth et al., 2024c). In the present study, we examined the SNARC effect across three cultures by using both PJ and MC tasks to reveal whether the cultural directionality hypothesis is applicable only when the magnitude is task-relevant (MC), or whether also applicable when the spatial representation of magnitude is not relevant, and automatically activated (PJ). The latter case would emphasize the strength of the influence of cultural directionalities on the SNARC effect independent from the task.

Concerning the strength of the SNARC effect in these tasks, the findings remain inconsistent. Some studies reported that the SNARC effect is more prominent in MC than in PJ (Cheung et al., 2015; Bae et al., 2009; Fitousi et al., 2009; van Dijck et al., 2009), others reported the reverse pattern; the SNARC effect is stronger in PJ than in MC (Georges et al., 2017; Gevers et al., 2010; Ito & Hatta, 2004) and Didino et al. (2019) reported no difference on the strength of the SNARC effect between the tasks. If the strength of the SNARC effect is related to the mean reaction times (longer RTs related to stronger SNARC; Cipora et al., 2019a, Supplementary Material; Didino et al., 2023; Gevers et al., 2010), we would expect the SNARC effect to be stronger in PJ than MC as RTs in the latter are shorter (Gevers et al., 2010; Wood et al., 2008). However, Wood et al. also reported that the strength of the SNARC effect cannot be only explained by the duration of responses but also by task demands (i.e., whether numbers need to be semantically processed in the task or not). We expected task-relevance of number magnitude (i.e., in MC but not in PJ) to more strongly influence the strength of the SNARC effect than overall RTs (i.e., faster responses in MC than in PJ) so that we hypothesized a stronger SNARC effect in MC than in PJ.

### MARC effect

Apart from number magnitude, number parity can also be mapped onto space. More specifically the *Linguistic Markedness of Response Codes* (MARC) effect (Nuerk et al., 2004), describes faster left/right responses to odd/even numbers, respectively. The activation of the parity of a number is not as automatic as the magnitude of the number, though, and therefore, the MARC effect typically only arises in PJ but not in MC.

Linguistic markedness refers to the characteristic of items in frequently used pairs (e.g., good-bad, right-wrong, even–odd, and left–right) that are typically associated with negative and positive polarities (see also Proctor & Cho, 2006). The marked member of the pair is typically more negative and vice versa (e.g., good-unmarked, bad-marked). Nuerk et al. (2004) suggested that the MARC effect is a congruity effect between the markedness of the parity of the number and the response hand (i.e., faster responses for congruent than incongruent situations). Since the MARC effect is influenced by linguistic experiences (e.g., in deaf German signers; Iversen et al., 2004, 2006) and individual differences (e.g., handedness; Huber et al., 2015), one might expect influences of culture as well. To the best of our knowledge, no MARC differences across different reading directions/cultures have been reported. Therefore, in the current study, we examined the MARC effect at the group level in PJ across all samples to examine the cultural influences on the MARC effect.

### Finger counting habits

Finger-counting habits seem to be related to the SNARC effect (Fischer & Brugger, 2011). Fischer (2008) reported that participants who count their fingers from the left hand, following an LR direction show a significant SNARC effect, whereas the ones who count from the opposite direction do not. Note that despite several investigations, the effect of finger counting on the SNARC effect is largely inconclusive (e.g., Fabbri, 2013; Hohol et al., 2022; Prete & Tommasi, 2020; Wasner et al., 2014). Lindemann et al. (2011) suggested that the finger-counting direction is consistent with the reading direction of cultures (as many cultural directional preferences). However, this observation has been questioned in later studies showing great within-culture variability in finger counting routines, and in several Western European countries, the majority of participants started from the right hand (e.g., French, Belgian, or Italian participants, see Hohol et al., 2018, for a brief overview). From these aspects, the influence of finger counting on the SNARC effect does not serve as an informative variable, though it could be an explanatory factor in the cultural influences on SNAs. Therefore, we explored finger counting habits and their influence on the SNARC effect across three cultures.

### Preregistered hypotheses

The hypotheses were preregistered at https://osf.io/pg5tn. First, we expected directional preferences in the CDPQ to be more LR in German compared to Turkish participants. Furthermore, a stronger RL preference was expected in Iranian compared to Turkish participants. Evidence for these hypotheses would support the cultural directionality hypothesis and our assumption that Turkish is a mixed culture in terms of directionalities (i.e., directional preferences and reading direction).

Furthermore, we expected a regular SNARC effect in German participants based on previous findings for both PJ and MC tasks (see Wood et al., 2008). On the other hand, we had no specific hypotheses for the group-level SNARC effect in Turkish and Iranian samples due to inconclusive or insufficient previous evidence.

Most importantly, we hypothesized a more prominent regular SNARC effect in the German compared to the Turkish sample. Evidence for this would suggest that cultural directional preferences influence SNAs beyond reading direction (which is from LR in both samples). On the other hand, based on the reading direction account, we hypothesized a more prominent SNARC effect in the Turkish compared to the Iranian sample. Evidence for this hypothesis would reveal an effect of reading (LR vs. RL) by reducing the involvement of cultural directional preferences, which we expect to be RL in both cultures. Therefore, we tested the German < Turkish < Iranian order for SNARC slopes.

We expected the SNARC effects to be more pronounced in the MC compared to PJ because the magnitude processing is task-relevant in MC, unlike PJ.

## Method

### Participants

A total of 1,144 individuals clicked on the study link. Only 476 of them (157 from Germany, 147 from Turkey, and 172 from Iran) started the experimental tasks and 412 (146 from Germany, 137 from Turkey, 129 from Iran) completed all the experimental tasks.

Participants were university students and recruited via university round mail and social media. Their participation was compensated with €5 or the approximate equivalent in Turkish and Iranian currency. The study was approved by the ethics committee of the Eberhard Karls University of Tuebingen’s Department of Psychology.

We applied the following exclusion criteria before the analysis. Unless otherwise noted, all the exclusion criteria below were preregistered. First, we excluded the datasets of 30 participants (5 from Turkey, and 25 from Iran) who reported not wanting to participate seriously but only out of curiosity (seriousness check; e.g., Reips, 2009).

Subsequently, the datasets of 5 participants from Germany who reported not being native German speakers were excluded. Also, there were some bilingual participants in the sample. Since our main hypotheses focus on the culture and reading direction of individuals, we excluded datasets from bilingual participants whose second language is written in reverse direction from their first (LR for Iran and RL for Germany and Turkey), or whose second language belongs to the Middle Eastern (for Germany) or Western culture (for Turkey and Iran). Based on this, the datasets were excluded from 14 bilingual participants (4 from Germany, 3 from Turkey, and 7 from Iran; see Table 1 in the Supplementary Material for detailed information about bilingual participants). This criterion was not preregistered. For the German, Turkish, and Iranian samples, we aimed to recruit participants who were residents of the respective countries. In the Iranian sample, data sets from two participants were excluded, one who reported living in the USA at the time of the experiment and one who reportedly spent the majority of their life in Turkey. These exclusion criteria were also not specified in the preregistration. Since the exclusion criteria for bilingual participants and location were not specified in the preregistration, we also provided the main findings by including datasets from these participants in the Supplementary Materials (Tables 9–14). There was no considerable change in the findings.

Also the datasets of 21 participants (2 from Germany, 7 from Turkey, and 12 from Iran) who described their environment as very/extremely noisy or reported multiple major distractions during the experiments were excluded. Finally, datasets of 23 participants (5 from Germany, 10 from Turkey, and 8 from Iran) were excluded due to having less than 75% valid trials remaining or leaving at least one empty experimental cell to calculate the SNARC effect (number magnitude * response side) in any of the tasks (see Data Preparation section below for further details).

During the preregistration phase, we planned to collect valid data from 130 participants from each sample. However, after applying all the exclusion criteria, the analysis was conducted with 130 German participants (90 women, 37 men, 3 unspecified; 119 right-handed, 8 left-handed, and 3 ambidextrous), 112 Turkish participants (71 women, 40 men, 1 other; 95 right-handed, 12 left-handed, and 5 ambidextrous), and 75 Iranian participants (15 women, 60 men; 63 right-handed, 11 left-handed). Six of the participants did not report their age. All of the remaining participants reported being at least 18 years old. The average age was 24.22 (range = 18—38 years, *SD* = 3.69) for German, 21.75 (range = 18—29 years, *SD* = 2.12) for Turkish, and 24.45 (range = 18—52 years, *SD* = 4.29) for Iranian participants.

Only three participants in the German and one in the Turkish sample reported being able to communicate in a foreign language with RL direction. Forty-six Iranian participants reported being able to communicate in a foreign language with LR direction. See Table 2 in the Supplementary Material for detailed information about the foreign languages that the participants speak. None of the participants’ datasets were excluded based on their foreign language.

### Materials and procedure

This study was preregistered on the Open Science Framework (OSF: 10.17605/OSF.IO/PG5TN) and administered in the native language of participants (i.e., German, Turkish, and Farsi). The data was collected online between April 11, 2023, and July 31, 2023, simultaneously in all countries. While there are technical and methodological issues with reliability in individual measurement of response times based on operating systems and web browsers used (e.g. Bridges et al., 2020; Garaizar & Reips, 2019), such variation in response times cannot systematically alter support for or against our hypotheses in the present study, as it follows an experimental design with randomization and technical variation over the Internet rather increases external validity compared to lab studies with a reduced set of instrumentation (Reips, 2002, 2021).

Participation was only possible via a desktop computer or laptop. The experimental procedure was implemented via JavaScript (88.1%), CSS (7.9%), HTML (3.3%), and PHP (0.7%) and administered online. An English demo version of the experimental procedure is available at https://osf.io/dk4we. At the beginning of the experiment, participants were asked to participate only if they were at least 18 years old. Furthermore, they were asked to perform the experiment in a quiet room and were informed that they could withdraw at any point by closing the browser tab.

After giving their informed consent, participants were first asked to indicate whether they wanted to seriously participate in the study. After that, there were some demographic questions about their gender, age, native and foreign languages, whether they are actively using their foreign languages, the weekly duration (hours/minutes) of the foreign language use in handwritten, typed, or spoken, country of residence, the country that they spent the majority of their life, familiarity with Western/Middle Eastern cultures, and their handedness. Next, they performed PJ and MC tasks. After the experimental tasks, they responded to the CDPQ, how they represent good and bad on the horizontal space, and finally, to the finger-counting assessment questions. These three parts were only assessed after the completion of the PJ and MC tasks, to avoid triggering a potentially associated spatial mapping. At the end of the study, participants were asked to report on the quietness of the environment and on any major distractions during the participation. English versions of all materials are available at https://osf.io/2w7jp.

#### PJ and MC tasks

In both PJ and MC tasks, the stimuli were eight numbers from 1 to 9 except 5. Arabic numerals (i.e., 1, 2, 3, 4, 6, 7, 8, and 9) were used for the German and Turkish experiments, whereas Eastern Arabic numerals (i.e., ١, ۲, ۳, ۴, ۶, ۷, ۸, and ۹) were used for the Farsi experiment. Stimuli were black (0, 0, 0, in RGB notation) on a gray (150, 150, 150, in RGB notation) background. Each trial started with a black square (extended ASCII 254, side length of 72px) for 300 ms as a fixation point. Then the number (Open Sans font, size 72px) was presented until a response was given. Finally, a blank gray screen (150, 150, 150, in RGB notation) for 500 ms concluded the trial.

Each number was presented 40 times in both tasks. Hence, each task consisted of 320 trials divided into two blocks (with a minimum 30-s break and a switch of response-to-key assignment in between) in which each number was presented 20 times in a randomized order. Participants responded to the parity status of the numbers during the PJ task (i.e., whether odd or even) and the magnitude of the numbers during the MC task (i.e., whether smaller or larger than 5) in a binary response setup. The response-to-key assignment was reversed between the blocks which resulted in a MARC-compatible (i.e., odd-left and even-right) and a MARC-incompatible (i.e., odd-right and even-left) block in PJ and a SNARC-compatible (i.e., small-left and large-right) and a SNARC-incompatible (i.e., small-right and large-left) block in MC. As a default, the D and K keys were defined as left and right response keys, respectively. These keys were in similar positions on keyboards that are the most common in the respective country (e.g., D is the third letter from the left and K is the fourth letter from the right on the middle row in all keyboards). Participants had the option to change these response keys to any other two keys that met the criteria of being in the same row and at least four keys’ width apart on their keyboard, but none took up this option. The order of the PJ and MC tasks was randomized between participants. Further, each participant was randomly assigned one of two different block orders (SNARC-/MARC-compatible first and SNARC-/MARC-incompatible second in both tasks or SNARC-/MARC-incompatible first and SNARC-/MARC-compatible second in both tasks). Each block was preceded by a short practice session of eight trials (each number presented once). During practice sessions, the required response-to-key assignment was given at the bottom of the screen, and participants received feedback on the accuracy of their responses. Practice sessions were followed by instruction re-iterating the response to the key assignment and the start of the experimental block. No feedback and prompts were presented during experimental blocks.

#### CDPQ

**Fig. 2:**
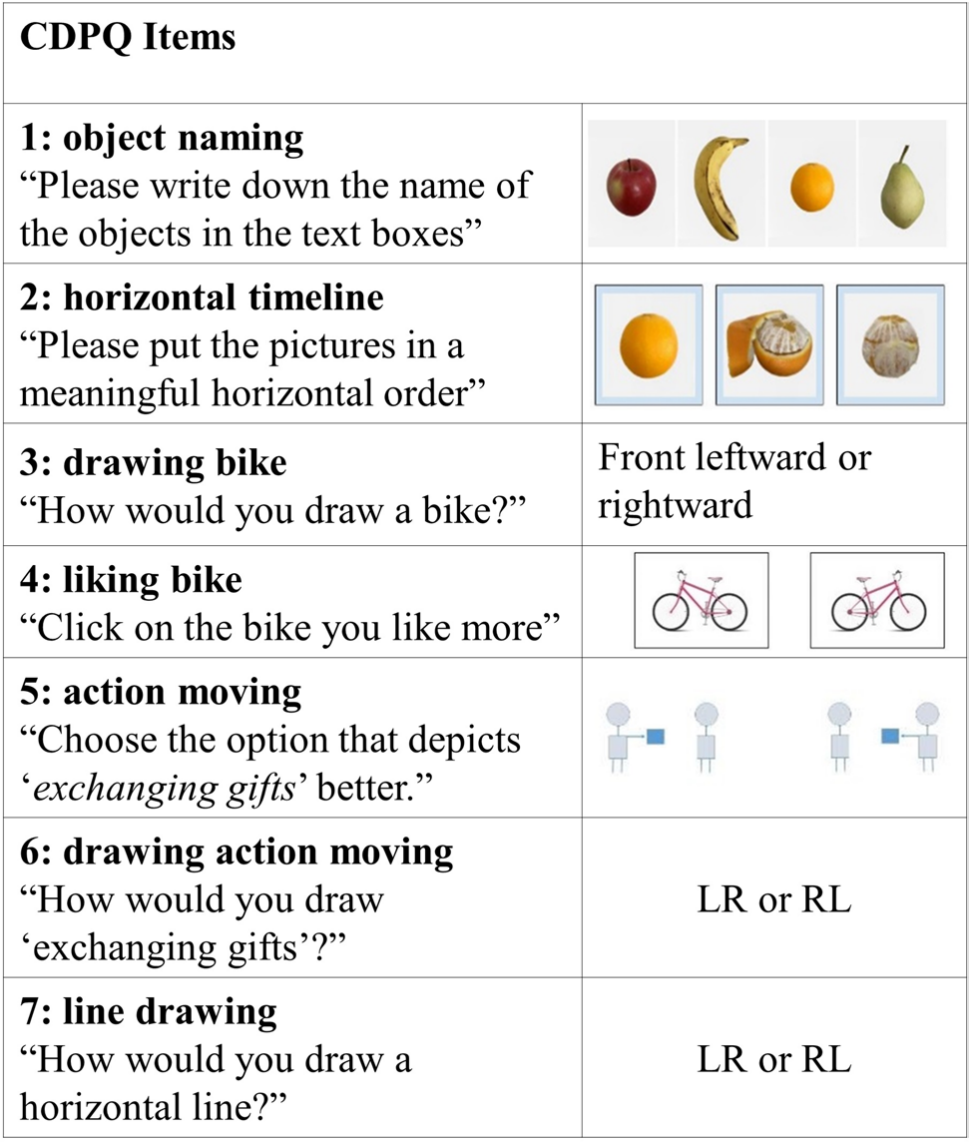
Items in CDPQ. *Bicycle depictions were designed by FreePik

The CDPQ included seven items (see Fig. 2). Item 1 was an object-naming task; participants wrote the names of the horizontally aligned fruit pictures in a vertical (top-down) order. We recorded whether participants followed the LR, RL, or mixed order. In Item 2, participants put three pictures of consecutive events (i.e., an orange being peeled) in a horizontal timeline (Tversky et al., 1991). We recorded whether participants organized the temporal preceding from left to right or right to left or in mixed order. In Items 3 and 4, participants were asked how they would draw a bicycle (leftward or rightward; Vaid, 1995) and to choose the bicycle they liked from two mirror-inverted pictures (one facing left and the other right or indicated that they do not have a preference). In Items 5 and 6, we asked participants how they would draw the scene “exchanging gifts between two people” (the giver on the left and the receiver on the right, vice versa, or no preference) (Maass & Russo, 2003), and to choose the option that depicts this scene better out of two mirror-inverted pictures (action moving from LR or RL). In Item 7, we asked participants to state whether they would draw a horizontal line from LR, RL, or did not have a preference (Lieblich et al., 1975). The presentation order of items was fixed among participants except that the order of Items 3–4 and 5–6 were switched randomly across participants. During the administration of most CDPQ items, participants first saw the instructional text, and only on the next page, they saw the pictures and respond (Items 1, 2, 4, and 5). This specific procedure was preferred to prevent the directional activity of instruction reading from inducing the preference. For items that did not include any pictures but instead only text (Items 3, 6, and 7), participants saw the question and answer options together and responded on the same page. The instructional text and pictures (for items with a picture) and questions and options (for items without a picture) remained on the screen until the participant responded. An English version of CDPQ is available at https://www.numcog.psychologie.uni-tuebingen.de/?demo&en&p=cdq_1.

Apart from the CDPQ, we also asked participants how they would associate the good and the bad with the horizontal space (see Supplementary Material for the findings on this question).

#### Finger counting assessment

The participants’ finger-counting behavior was measured with two questions. First, “How do you count your fingers from 1 to 10?” for which the responses were “I prefer to start counting with the fingers of my right hand”, “I prefer to start counting with the fingers of my left hand”, “I do not know or I do not have any preferred hand”, or “I prefer not to answer”. The second question was “How stable are your finger-counting habits?” and the responses were “I always start counting with this hand”, “I usually start counting with this hand”, “I do not know or I do not have a preferred hand or stable habit”, or “I prefer not to answer”.

### Analyses

In addition to all the frequentist tests described below, we report corresponding Bayes Factors to differentiate evidence of the absence of an effect from the absence of evidence for an effect. To calculate Bayes Factors, we used the default r-scale of 0.707 as an uninformed prior in the Cauchy distribution. To interpret Bayes Factors, we follow Dienes’ (2021) recommendations, namely, a BF_10_ above 3 or 10 will be treated as moderate or strong evidence for the alternative hypothesis, and a BF_10_ below 1/3 or 1/10, as moderate or strong evidence for the null hypothesis.

#### PJ and MC tasks

##### Data preparation

All steps of the data preparation followed our preregistered protocol. Only experimental trials were analyzed. First, incorrect trials were excluded (4.92% in PJ and 3.67% in MC). Next, RTs shorter than 200 ms and longer than 1500 ms were discarded (1.69% in PJ and 1.13% in MC). Later, within each participant, a sequential filtering approach (Cipora & Nuerk, 2013; Cipora et al., 2019a) was applied to trim outlier RTs in the data separately for PJ and MC tasks. Specifically, RTs outside ± 3 SD from the individual’s mean RT were excluded. This filtering procedure was repeated until means and SDs no longer changed, resulting in an exclusion of 4.15% of the data in PJ and 4.97% of the data in MC. For 23 participants (5 from the German, 10 from the Turkish, and 8 from the Iranian sample), more than 25% of the dataset was discarded in at least one of the tasks, and these participants’ data were excluded from the analysis. This left data from 130 German, 112 Turkish, and 75 Iranian participants for further analysis. After RT preprocessing, 89.24% of this data in PJ and 90.23% of their data in MC were retained.

##### Data analyses

All the data analyses described here were performed by following the preregistration unless otherwise stated. In all analyses, a significance level of ɑ = 0.05 was used. We had not preregistered how to correct ɑ for multiple tests. Bonferroni-Holm method was applied to correct for multiple tests in all analyses unless noted otherwise.

We investigated the SNARC effect in both PJ and MC tasks, and the MARC effect only in the PJ task, by using a repeated-measures regression approach (proposed by Lorch & Myers, 1990; adapted to the SNARC effect by Fias et al., 1996). First, dRTs (mean right-hand response RT minus mean left-hand response RT) were calculated for each number separately within each task for each participant.

Based on the continuous shape of the SNARC effect in PJ (Wood et al., 2008), a linear predictor was defined with the values 1, 2, 3, 4, 6, 7, 8, and 9. Furthermore, the parity of numbers was added to the regression model of the PJ task as a contrast-coded predictor (with -0.5 for odd and 0.5 for even numbers) to investigate the MARC effect (see Nuerk et al., 2004, 2005; Cipora et al., 2019a). Number parity is orthogonal to the number magnitude in the given stimuli set, therefore, including the parity as an additional predictor in the regression model does not affect the parameter estimate of number magnitude (Cipora et al., 2019a).

For the MC task, average dRTs were calculated for small (1, 2, 3, and 4) and large (6, 7, 8, and 9) numbers. Based on the categorical shape of the SNARC effect in MC tasks (Wood et al., 2008), a categorical predictor was defined with the values -0.5 for small numbers and + 0.5 for large numbers.

The resulting coefficients for number magnitude from the individual analyses were considered as unstandardized SNARC slopes. In the PJ task, negative unstandardized SNARC slopes reflect the increase in RT advantage (in milliseconds) of the right hand over the left per increase in number magnitude in one unit (because number magnitude is a linear predictor). Coefficients for the number parity predictor were considered as unstandardized MARC slopes. Negative unstandardized MARC slopes reflect the increase of the RT advantage of the right hand over the left hand for even numbers compared to odd numbers. In the MC task, negative unstandardized SNARC slopes reflect the increase of the RT advantage of the right hand over the left hand for large numbers over small numbers.

In addition, standardized SNARC and MARC slopes (Fisher-z-transformed standardized regression weights) were calculated (see Hochmann et al., 2024; Cipora et al., 2019a). Standardized slopes can be considered as the individual effect sizes of the SNARC and MARC effects and are not influenced by individual reaction time or individual variance within reaction time (Cipora et al., 2019b). More negative slopes for both magnitude and parity predictors correspond to the stronger SNARC and MARC effects, respectively.

Unstandardized and standardized SNARC and MARC slopes were compared against zero using two-sided one-sample *t*-tests to examine the group-level SNARC and MARC effects. The task order (PJ first and MC first) and block order (SNARC-/MARC-compatible first and SNARC-/MARC-incompatible first) effects were examined using independent-sample *t*-tests in each sample (see Supplementary Material Tables 4 and 5 for the findings of this analysis).

Subsequently, unstandardized SNARC slopes were compared across samples with a Jonckheere-Terpstra test for monotone trend with 2000 permutations, one-sided for the German < Turkish < Iranian alternative hypothesis. This nonparametric test, in comparison to linear contrast in ANOVA, only assumes a monotone trend in the data, while it does not assume equidistance between group means. Therefore, it best reflects our hypothesis as we could not assume that the German vs. Turkish mean difference would be the same as the Turkish vs. Iranian difference.

This was followed by comparison of the PJ and MC in terms of the size of the unstandardized SNARC effect. It is not possible to compare a categorical and a linear slope because the former is naturally much larger when considering the same underlying dataset, therefore, additional individual regression analyses were performed for the MC task with magnitude being a linear predictor just as for the PJ task. Then the unstandardized SNARC slopes from the continuous models were compared with paired samples *t*-tests within each sample. Moreover, to reveal a possible relationship between the SNARC effects observed in the two tasks, the correlation of the unstandardized SNARC slopes between the tasks (continuous for parity judgment and categorical for magnitude classification) was examined across samples.

Furthermore, H0 bootstrapping analysis (Cipora et al., 2019b; see also Roth et al., 2024b) was performed to calculate the individual prevalence of SNARC and MARC effects within each sample. The analysis calculates the probability of finding the SNARC/MARC slopes as we observed during the experiments for each participant, assuming there was no association between magnitude/parity of the number and response side (i.e., if the null hypothesis was true). Separately for the tasks, within each individual’s dataset, two sets of 20 responses were randomly sampled with replacement from all RTs to a given number. One of the sets was considered to be left-hand responses and the other, right-hand responses. Next, unstandardized SNARC and MARC slopes were calculated using these sampled data. This procedure was repeated 5000 times, resulting in a bootstrapped slope distribution for each participant. Next, empirically obtained slopes were examined to see whether they were outside or inside of the mid-90% of bootstrapped slope distributions (i.e., 90% H0 CIs). Based on these CIs, the individual slopes were classified as reliable SNARC or MARC effect (empirically obtained slope < lower bound of H0 CI), reliable reverse SNARC or MARC effect (empirically obtained slope > upper bound of H0 CI), or unreliable SNARC or MARC effect (empirically obtained slope within H0 CIs; see Hohol et al., 2020, 2022; van Dijck et al., 2022). A *χ*^*2*^ test was calculated to investigate whether the proportions differ between samples.

We examined the impact of foreign languages (RL for German and Turkish and LR for Farsi) on the SNARC effect by correlating the duration (in minutes) of the foreign language use (handwritten, typed, or spoken) and the unstandardized SNARC slopes from the two tasks (see Supplementary Material for the findings of this analysis).

#### CDPQ

For each item, a 3 (sample: German, Turkish, and Iranian) × 3 (directionality: LR, NP (no preference), and RL) *χ*^*2*^analysis was performed. Fisher’s Exact Test was performed instead of a *χ*^*2*^ when expected frequencies in any of the cells were lower than 5. In addition to the descriptive analyses, a sum score was calculated for each participant for the CDPQ. First, each LR response was scored as -1, and each RL response as 1. If there was no LR or RL order in the first two items (i.e., object naming task and timeline), these responses were scored as 0. In Items 3–7, “I have no clear preference” responses were also scored as 0. Therefore, an individual sum score across the seven items could vary between -7 (very strong LR preference) and 7 (very strong RL preference). The participants’ sum score was compared across samples with a one-sided Jonckheere-Terpstra test for monotone trend with 2000 permutations for the alternative hypothesis German < Turkish < Iranian. Finally, the correlation between the unstandardized SNARC slopes and the sum scores of the CDPQ was examined across samples.

#### Finger counting

Independent samples *t*-tests were performed in each sample to examine the possible effect of finger counting direction (left- vs. right-starters) on the unstandardized SNARC slopes. Furthermore, a Jonckheere-Terpstra test for monotone trend with 2000 permutations was performed separately for left- and right-starters in each sample to reveal whether finger counting stability has any influence on the unstandardized SNARC slopes (see Supplementary Material Tables 7 and 8 for the findings of this analysis).

## Results

### The SNARC effect and the MARC effect

**Fig. 3:**
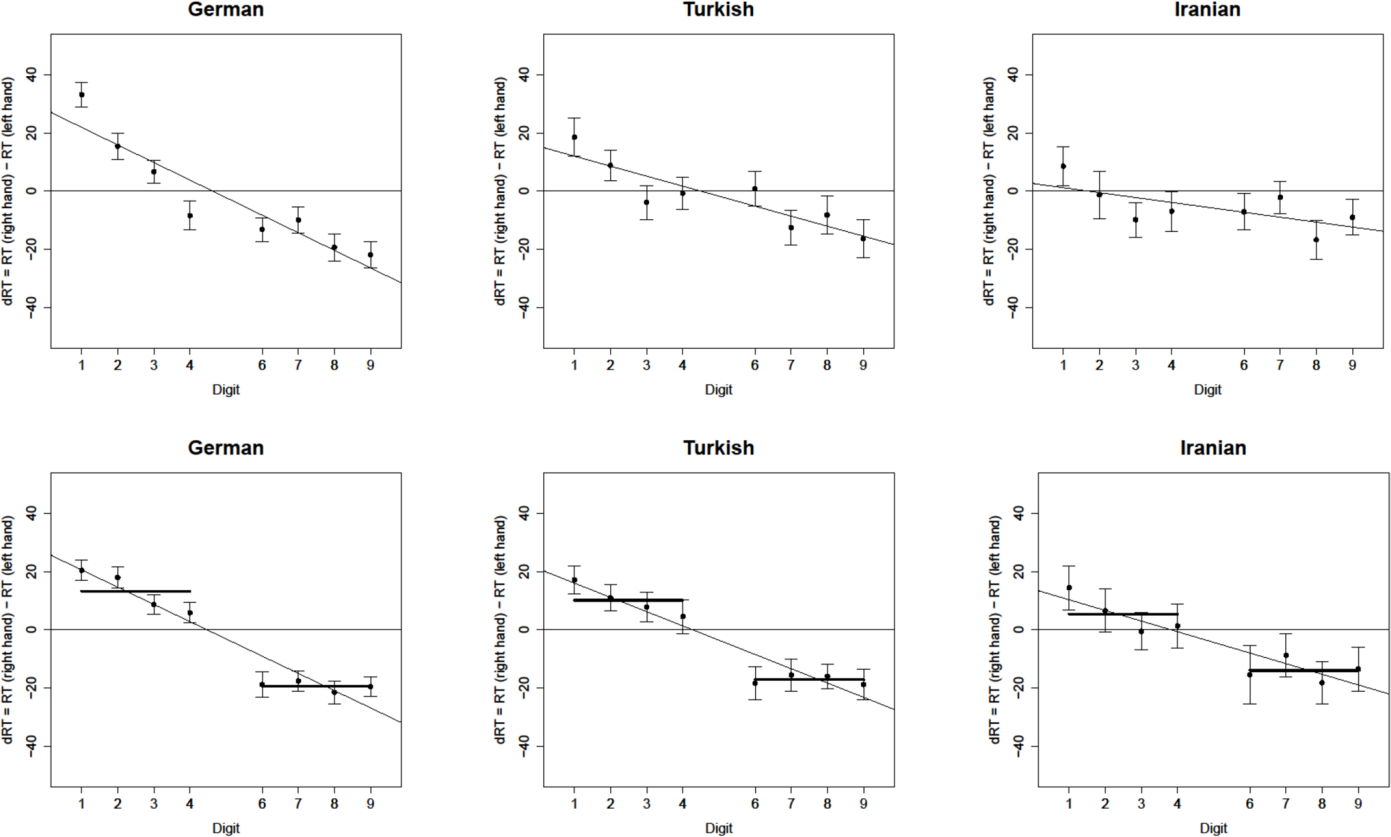
The SNARC slopes in PJ (upper panel) and MC (lower panel) across three samples. The SNARC effect is characterized by a negative slope, indicating larger digits were responded to faster with the right than with the left-hand

**Fig. 4:**
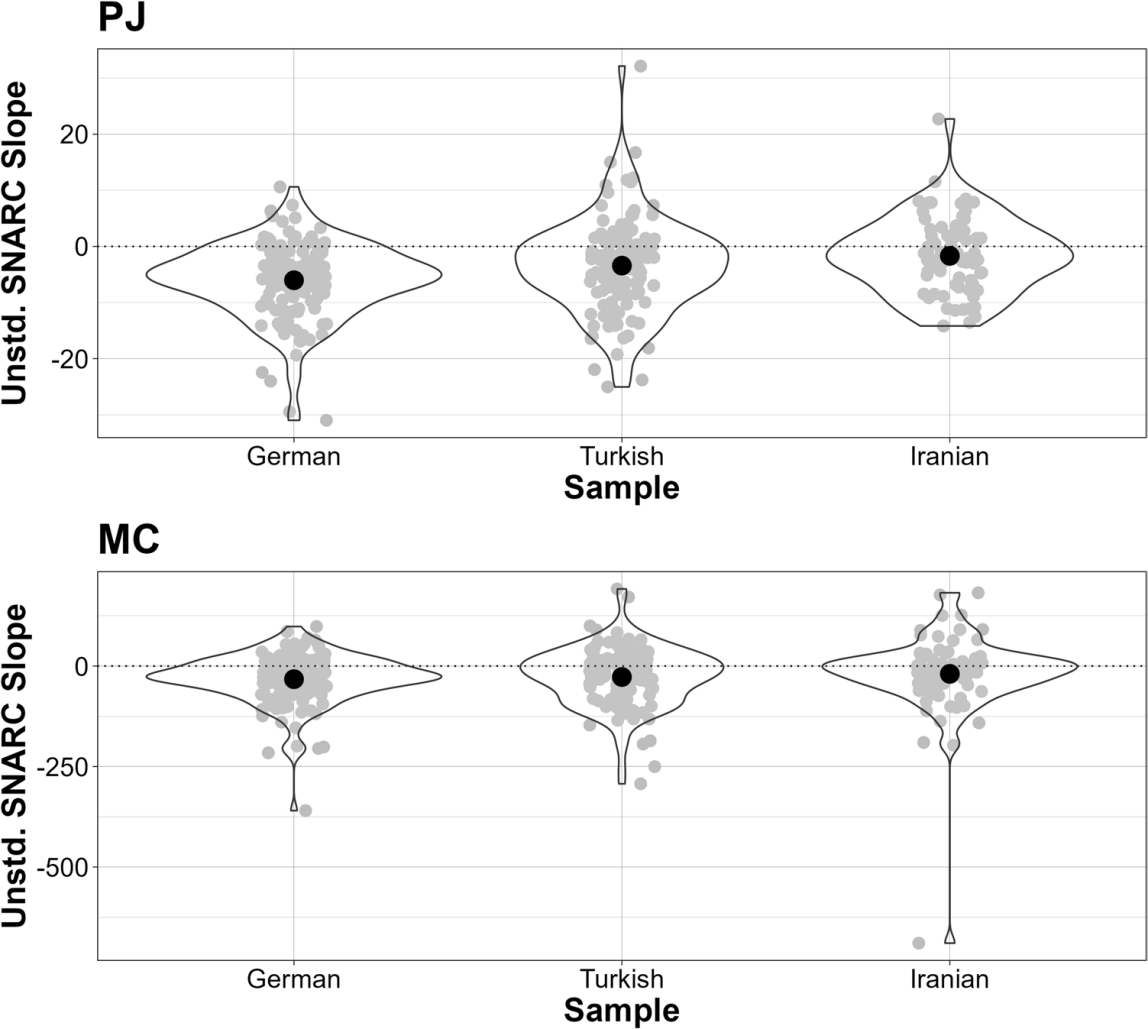
The distribution of the unstandardized SNARC slopes in each sample

The results of all the *t*-tests performed to investigate the presence of the SNARC and the MARC effect across all samples in the PJ task are shown in Table 1. There was a significant SNARC effect at the group level across all samples (Fig. 3). Note that the Bayesian *t*-test showed inconclusive evidence regarding both the unstandardized and the standardized SNARC effect in the Iranian sample. The distribution of the unstandardized SNARC slopes for each sample can be seen in Fig. 4. Interestingly, no significant unstandardized or standardized MARC effects were observed in any of the samples.

**Table 1:** The SNARC effect and the MARC effect in PJ

Measure	Sample	Mean Slope (SD)	*t*	*p*	BF_10_
SNARC	German	−6.03 (6.68)	−10.30	< 0.001*	3.33e + 15*
	Turkish	−3.43 (8.63)	−4.21	< 0.001*	319.74*
	Iranian	−1.69 (6.72)	−2.18	0.033*	1.16
Standardized SNARC	German	−0.43 (0.45)	−10.72	< 0.001*	3.50e + 16*
	Turkish	−0.21 (0.46)	−4.79	< 0.001*	2773.61*
	Iranian	−0.11 (0.48)	−2.03	0.045*	0.89
MARC	German	−8.39 (68.36)	−1.40	0.164	0.25*
	Turkish	3.71 (83.44)	0.47	0.639	0.12*
	Iranian	−4.92 (72.57)	−0.59	0.559	0.15*
Standardized MARC	German	−0.14 (0.83)	−1.90	0.060	0.56
	Turkish	0.04 (0.84)	0.45	0.656	0.12*
	Iranian	−0.10 (0.81)	−1.06	0.293	0.22*

All tests were two-sided and against zero

*df* was 129 for the German, 111 for the Turkish, and 74 for the Iranian sample

^*^ *p* < 0.05 indicating a significant finding (frequentist), BF_10_ < 1/3 indicating a conclusive finding for the null hypothesis, and BF_10_ > 3 indicating a conclusive finding for the alternative hypothesis (Bayesian)

The results of the *t*-tests performed to investigate the presence of the SNARC effect across all samples in the MC task are shown in Table 2. There was a significant SNARC effect at the group level in German and Turkish samples with unstandardized and standardized measures, but no significant unstandardized or standardized SNARC effects in the Iranian sample. Furthermore, Bayesian *t*-tests showed inconclusive evidence for the alternative hypothesis of the presence of the unstandardized and standardized SNARC effect in the Iranian sample.

**Table 2:** The SNARC effect in MC

Measure	Sample	Mean Slope (SD)	*t*	*p*	BF_10_
SNARC	German	−32.58 (63.38)	−5.86	< 0.001*	299,484.60*
	Turkish	−27.26 (73.84)	−3.91	< 0.001*	113.51*
	Iranian	−19.42 (104.92)	−1.60	0.113	0.43
Standardized SNARC	German	−0.34 (0.61)	−6.31	< 0.001*	3.50e + 16*
	Turkish	−0.23 (0.59)	−4.04	< 0.001*	2773.61*
	Iranian	−0.13 (0.60)	−1.83	0.072	0.89

All tests were two-sided and against zero

*df* was 129 for the German, 111 for the Turkish, and 74 for the Iranian sample

^*^p < 0.05 indicating a significant finding (frequentist), BF_10_ < 1/3 indicating a conclusive finding for the null hypothesis, and BF_10_ > 3 indicating a conclusive finding for the alternative hypothesis (Bayesian)

### Comparing the SNARC effect across samples

As expected, the one-sided Jonckheere-Terpstra test (2000 permutations) revealed a significant monotone trend of German < Turkish < Iranian SNARC slopes in PJ, *T*_*JT*_ = 19,905, *p* < 0.001. One-sided independent samples Welch *t*-test showed a significant difference for the SNARC slopes of German and Turkish samples (*t*(207.48) = -2.59, *p* = 0.005) but not for the Turkish and Iranian samples (*t*(180.75) = -1.55, *p* = 0.061). Comparing the SNARC slopes with independent Bayesian post-hoc *t*-tests provided moderate evidence for a stronger SNARC effect in the German than in the Turkish sample (BF_10_ = 3.68), but evidence in the Turkish-Farsi comparison remained inconclusive (BF_10_ = 0.44).

Similarly, the one-sided Jonckheere-Terpstra revealed a significant result for the directional alternative hypothesis of German < Turkish < Farsi SNARC slopes in MC, *T*_*JT*_ = 17,835, *p* = 0.041. One-sided independent samples Welch *t*-test showed no significant difference between German-Turkish (*t*(220.26) = -0.60, *p* = 0.276) and Turkish-Farsi (*t*(122.27) = -0.56, *p* = 0.288) comparison on the SNARC slopes. Comparing the SNARC slopes with independent Bayesian *t*-tests revealed moderate evidence for the null hypothesis in the German-Turkish comparison (BF_10_ = 0.17) and Turkish-Farsi comparison (BF_10_ = 0.19).

### Comparing PJ and MC tasks

Calculating the SNARC slopes of the MC task with the magnitude being a continuous predictor yielded similar findings to the analysis in which the magnitude was a categorical predictor. With the continuous predictor, for the German and Turkish samples, there was a significant SNARC effect at the group level (German: *t*(129) = -6.36, *p* < 0.001, *M* = -5.92, *SD* = 10.61; Turkish: *t*(111) = -4.22, *p* < 0.001, *M* = -4.90, *SD* = 12.30) but not for the Iranian group (*t*(74) = -1.81, *p* = 0.074, *M* = -3.65, *SD* = 17.46). Note that categorical predictors typically fit the MC task data better than continuous (Wood et al., 2008). In the present findings for German and Turkish data, the categorical predictor (adj. *R*^*2*^ was 0.92 and 0.93, respectively) was slightly a better fit than the continuous (adj. *R*^*2*^ was 0.91 and 0.90, respectively) in MC. Interestingly though, continuous (adj. *R*^*2*^ = 0.83) was a better fit than categorical (adj. *R*^*2*^ = 0.77) for Iranian data in MC. As can be seen in the upper panel of Fig. 3, this finding was due to the first four numbers, but not the larger numbers, being continuously arranged in dRTs.

The SNARC slopes of the PJ and MC obtained from continuous magnitude predictors were compared separately in each sample, and results show no significant difference between PJ and MC slopes in any of the samples (German: *t*(129) = 0.10, *p* = 0.918, BF_10_ = 0.10; Turkish: *t*(111) = -1.00, *p* = 0.318, BF_10_ = 0.17; Iranian: *t*(74) = -0.94, *p* = 0.353, BF_10_ = 0.19).

### Correlation between the PJ and MC SNARC slopes

There was no significant correlation between the PJ and MC unstandardized SNARC slopes in any of the samples (German: *r* = -0.02, *p* = 0.822, BF_10_ = 0.21; Turkish: *r* = -0.08, *p* = 0.386, BF_10_ = 0.31; Iranian: *r* = 0.10, *p* = 0.402, BF_10_ = 0.37).

### H0 bootstrap analysis

Table 3 summarizes the individual prevalence of the SNARC effect in all samples for both tasks. For the unstandardized SNARC effect in PJ, in the German sample, the majority showed a reliable regular SNARC effect, unlike in the Turkish and Iranian samples. A higher number of participants showed an unreliable regular or reversed SNARC effect than those who showed a reliable SNARC effect. A *χ*^*2*^ analysis confirmed a significant association between the culture and the number of participants who showed a reliable SNARC effect, *χ*^*2*^(4) = 18.67, *p* < 0.001.

**Table 3:** Individual prevalence of the reliable SNARC effect calculated with H0 bootstrapping in PJ and MC

Task	Sample (N)	Reliable SNARC (*N*)	Reliable reverse SNARC (*N*)	Unreliable SNARC (*N*)
PJ	German (130)	56.92% (74)	4.62% (6)	38.46% (50)
	Turkish (112)	37.50% (42)	11.61% (13)	50.89% (57)
	Iranian (75)	29.33% (22)	12.00% (9)	58.67% (44)
MC	German (130)	56.92% (74)	16.15% (21)	26.92% (35)
	Turkish (112)	46.43% (52)	19.64% (22)	33.93% (38)
	Iranian (75)	38.67% (29)	21.33% (16)	40.00% (30)

For the unstandardized SNARC effect in MC, the number of participants in the German and Turkish samples who showed a reliable regular SNARC effect was higher than those who showed an unreliable regular or reversed SNARC effect. On the other hand, in the Iranian sample, there was a more even balance between those showing unreliable and reliable regular SNARC effects. The *χ*^*2*^ analysis revealed no significant association between the culture and the number of participants who showed reliable SNARC effect in the MC task, *χ*^*2*^(4) = 6.86, *p* = 0.144.

Table 4 summarizes the individual prevalence of the MARC effect in all samples for the PJ task. The reliable regular MARC effect was more prominent in the German sample compared to the other samples. The *χ*^*2*^ analysis revealed no significant association between the sample and the number of participants showing a reliable MARC effect in the PJ task, *χ*^*2*^(4) = 8.67, *p* = 0.070.

**Table 4:** Individual prevalence of the reliable MARC effect calculated with H0 bootstrapping in PJ

Sample (*N*)	Reliable MARC (*N*)	Reliable reverse MARC (*N*)	Unreliable MARC (*N*)
German (130)	41.54% (54)	23.85% (31)	34.62% (45)
Turkish (112)	35.71% (40)	37.50% (42)	26.79% (30)
Iranian (75)	36.00% (27)	22.67% (17)	41.33% (31)

### Finger counting habits

In the German and Iranian samples, most participants reported counting their fingers starting from the right hand; interestingly, the majority of the participants in the Turkish sample reported starting from the left hand (see Supplementary Material Table 6). Most of the participants reported that they are always or usually stable in their finger-counting habits (see Supplementary Material Table 7). Unstandardized SNARC slopes of PJ and MC did not differ between left- and right-starters in any of the samples, with moderate Bayesian evidence indicating that finger-counting direction had no influence on the SNARC effect (see Table 5).

**Table 5:** Two-sided independent-sample t-test findings comparing the unstandardized SNARC slope of left- and right-starters

Sample	Task	*t*	*df*	*p*	BF_10_
German	PJ	0.87	119.88	0.389	0.27*
	MC	−1.08	117.09	0.283	0.33*
Turkish	PJ	−0.46	70.07	0.644	0.24*
	MC	0.25	86.67	0.801	0.22*
Iranian	PJ	−0.20	33.71	0.841	0.28*
	MC	0.59	36.50	0.562	0.31*

^*^ BF_10_ < 1/3 indicating a conclusive finding for the null hypothesis

Furthermore, a series of two-sided Jonckheere-Terpstra tests showed no difference in unstandardized SNARC slopes based on the finger counting stability for either left- or right-starters (see Supplementary Material Table 8).

### CDPQ

**Fig. 5:**
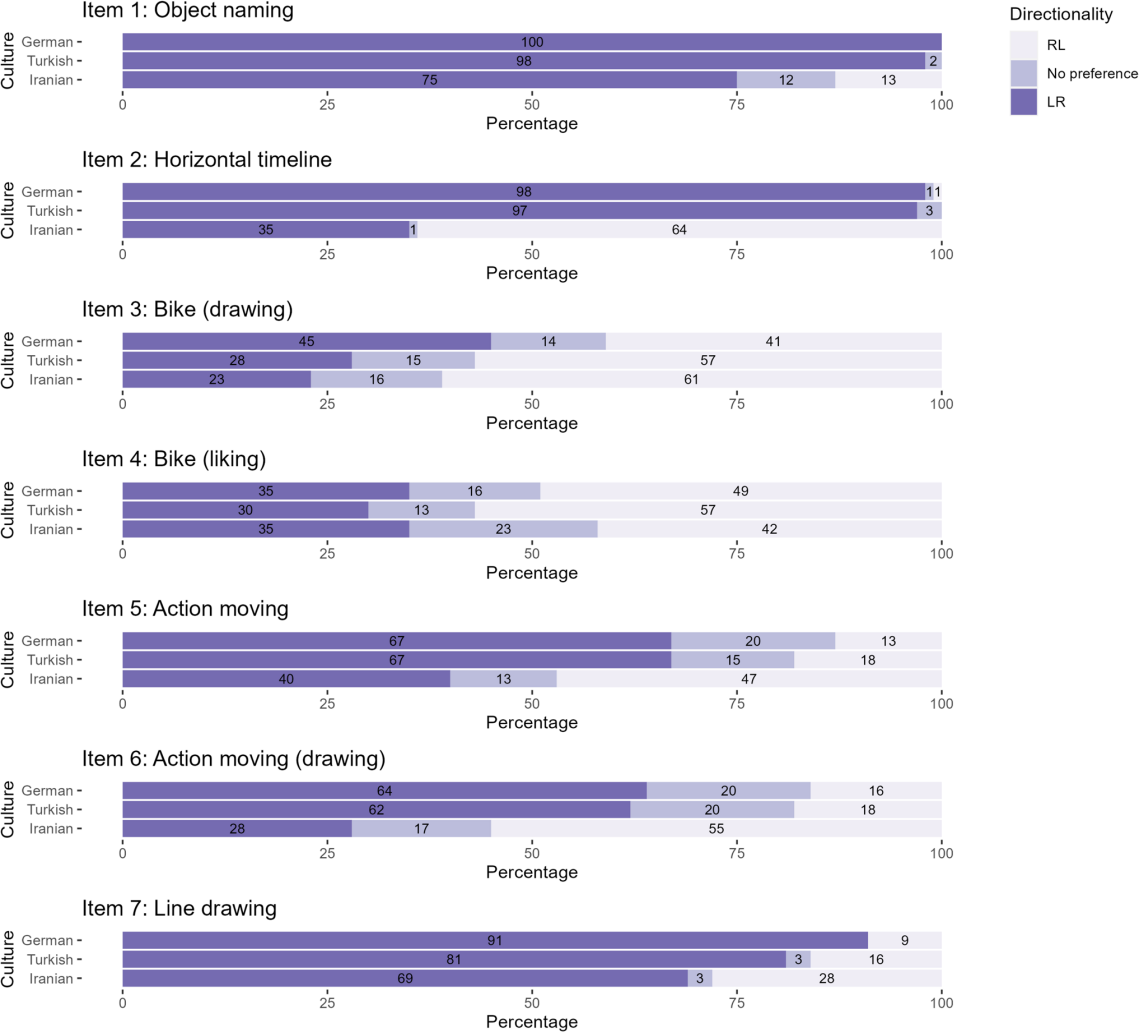
Directional Preferences in CDPQ Items

Figure 5 summarizes the directional preferences in CDPQ items. *χ*^*2*^ analysis performed for each item is shown in Table 6. Interestingly, samples did not differ based on liking a leftward or rightward-facing bicycle, although they showed differences in their preferences for drawing a bike. Post-hoc comparisons of samples for each item can be found in Tables 7 and 8.

**Table 6:** Χ^2^ analysis findings for each item in the CDPQ

**Items**	*χ*^*2*^	*df*	*p*
**1**: object naming^a^	–	-	< 0.001*

**2**: horizontal timeline^a^	-	-	< 0.001*
**3**: drawing bicycle	13.31	4	.010*
**4**: liking bicycle^b^	4.88	4	.300
**5**: action moving	33.80	4	< 0.001*
**6**: action drawing	44.59	4	< 0.001*
**7**: line drawing^a^	-	-	< 0.001*

^a^Fisher’s Exact test was performed when the expected frequencies of a cell were lower than 5

^b^Bicycle demonstration was designed by FreePik

^*^p < .05 indicating a significant finding

**Table 7:** Fisher’s Exact Test post-hoc comparisons for CDPQ items

Item	Comparison	p
1: object naming	German- Iranian	< 0.001*
	Turkish-Iranian	< 0.001*
	German- Turkish	.213
2: horizontal timeline	German- Iranian	< 0.001*
	Turkish-Iranian	< 0.001*
	German- Turkish	.820
7: drawing a line	German- Iranian	< 0.001*
	Turkish-Iranian	.118
	German- Turkish	.044*

Bonferroni correction was applied in post-hoc tests

^*^ *p* < 0.05 indicating a significant finding

**Table 8:** Χ^2^ analysis significant post-hoc findings

Item	Sample	Directionality	p
3: bike (drawing)	German	LR	< 0.01 (higher)*
		RL	< 0.01 (lower)*
5: action moving	German	RL	< 0.001 (lower)*
	Iranian	RL	< 0.001 (higher)*
		LR	< 0.001 (lower)*
6: action moving (drawing)	German	RL	< 0.001 (lower)*
	Iranian	RL	< 0.001 (higher)*
		LR	< 0.001 (lower)*

Only cells that are significantly lower or higher than expected frequencies were summarized in the table

Bonferroni correction was applied in post-hoc tests

^*^p < 0.05 indicating a significant finding

### CDPQ total score

In line with the unstandardized SNARC slopes, the one-sided Jonckheere-Terpstra test (2000 permutations) revealed a significant monotone trend of German < Turkish < Iranian total CDPQ scores, *T*_*JT*_ = 24,156.50, *p* < 0.001, indicating that LR directionality was decreasing in a monotone way from German (*M* = -3.65) through Turkish (*M* = -2.96) to the Iranian sample (*M* = 0.07).

While the between-group differences were significant, the correlations between the participants’ unstandardized SNARC slopes and the participants’ total CDPQ scores were not significant in any of the tasks or within any of the samples (PJ: German: *r* = -0.07, *p* = 0.435, BF_10_ = 0.27; Turkish: *r* = -0.06, *p* = 0.549, BF_10_ = 0.26; Iranian: *r* = -0.06, *p* = 0.617, BF_10_ = 0.30; MC: German: *r* = 0.21, *p* = 0.015, BF_10_ = 3.47, *adj. p* = 0.089; Turkish: *r* = 0.06, *p* = 0.542, BF_10_ = 0.26; Iranian: *r* = -0.10, *p* = 0.371, BF_10_ = 0.38). However, for Germans in the MC, the BF revealed anecdotal evidence, and frequentist testing would have been significant if tested one-sided (which was not specified as such in the preregistration). This provides a hint that the relationship between cultural directional habits and SNARC slopes may deserve more investigation in the future. On the other hand, given the relatively inconclusive results, we cannot draw strong conclusions from this data.

## Discussion

### Overview

The present study tested the cultural directionality hypothesis that SNAs are influenced by individuals’ socio-cultural environment, including reading direction and many other common cultural directional preferences. To test this hypothesis, we measured the SNARC effect with PJ and MC tasks in three different cultures; a predominantly LR (German), a predominantly RL (Iranian), and a mixed (Turkish) culture regarding cultural directional preferences and reading habits. After following an RL script for almost 600 years, Turkey changed to an LR script direction as recently as 100 years ago. We hypothesized that, despite this change, some cultural directional preferences prevailed in RL due to six centuries of that tradition. To confirm that cultural directional preferences are indeed more RL in Turkish culture compared to German and more LR compared to Iranian culture, we administered the newly developed CDPQ. Findings supported this categorization, the CDPQ total score being more strongly LR for German, more strongly RL for Iranian, and in between for Turkish participants. Results of the PJ and MC tasks were in line with CDPQ findings, showing that SNARC effects in both tasks were strongest for German, weakest for Iranian, and intermediate for Turkish participants. However, this group-level effect was not reflected in individual-level correlations. Overall, findings showed that directional preferences differed between cultures and the emergence of adult SNAs in two tasks was consistent with those cultural directional preferences.

### Cultural directional preferences across countries

As expected, the CDPQ showed that preferences were generally LR in German; however, surprisingly, they were not generally RL in Iran (see Faghihi & Vaid, 2023 for a review), but rather, indicating no preference overall. We could conceive two possible explanations for this.

First, it has been argued that LR directionality is initially rather innate (e.g., Felisatti et al., 2020) and only later overwritten by culture (e.g., McCrink & de Hevia, 2018). It is possible that this overwriting occurs only partially so that responses in the CDPQ are influenced by both innate LR directionality and cultural RL directionality, leading to an actual null effect. Note in this respect that the LR/RL directionality for Iranians differs greatly depending on the focus of attention. While object naming and line drawing were from LR, even in most Iranian participants, the action moving items, horizontal timeline, and bicycle (drawing) were predominantly from RL. Thus, it is possible that certain activities are more influenced by culture, and others, are less so.

Second, the world culture portrayed in movies and video games, experienced in international airports, computers, and mobile phone systems comes predominantly from LR cultures (e.g., the US). Individuals exposed to it are more likely to be confronted with LR culture in images and experience, which was the subject of our CDPQ. This global LR directionality might influence cultural habits in addition to their own culture. Some cultural aspects might be more RL because they are influenced more by their own culture (e.g., rarely encountered via international media, travel, and technology), while those more frequently encountered in international culture may be more LR. Since global culture and language influence many aspects of national languages (e.g., via loan words) and culture (e.g., via international music), it is clearly conceivable that this holds for directionality as well. It is therefore impossible to distinguish the two hypotheses in urban samples exposed to global culture, and future research should disentangle these by studying remote populations with RL culture and restricted or no access to global culture.

Overall, the preferences of German and Iranian participants for most items were in line with the previous findings of LR and RL cultures, respectively. The horizontal timeline and action moving items generally followed the respective culture direction as demonstrated previously (see Tversky et al., 1991; Mass & Russo, 2003). In the bicycle drawing, German participants reported a greater preference to draw the front-facing leftward compared to other samples. When the front is leftward, the bicycle appears moving RL, which might seem counterintuitive for an LR culture. On the other hand, this finding is in line with previous research with adults and children that demonstrated the tendency to draw the objects so that they appear moving RL in LR cultures, and so that they appear moving LR in RL cultures (Kebbe & Vinter, 2013; Vaid, 1995). The significant difference between samples in bicycle drawing disappeared in the expression of bicycle liking, suggesting that this directional bias difference across samples was specific to drawing habits, but not necessarily generalized to liking for objects. One possible explanation of this discrepancy between drawing and liking on directional preferences is that since people tend to start from the left side of the paper in LR cultures, they might place the most important and detailed part (i.e., the front) on the left side and continuing the drawing (i.e., the back) toward the right side. Correspondingly, we would expect the exact opposite pattern in RL cultures. Note that, in the present study, participants did not actually draw but only reported how they would draw the bicycle, which might underestimate their directional bias compared to lab studies. Interestingly, there was an overall LR preference in the object naming, including for Iranian participants. This finding is interesting since Shaki et al. (2012) showed RL preference in a similar task (i.e., object counting) in RL cultures (see Shaki & Fischer, 2023 for the influence of object counting direction on the attentional SNARC effect). On the other hand, a study by Shaki and Gevers (2011) suggested that cultural characteristics can manifest themselves in reverse directions for magnitude and ordinal information processing. In their study, magnitude information was associated with LR processing but ordinal information was with RL processing in Hebrew participants. In addition to that, although the tasks in the mentioned studies seem superficially similar, object naming in the present study and object counting in Shaki et al. (2012) might include different directionalities: object counting requires active object numbering by pointing at objects, whereas in the present study, participants identified pictures of the objects on a computer screen and wrote the words for each object, an activity which might be influenced by LR computer screen scanning habits on a more global scale. Although significantly less prominent in Iranian participants, there was an overall LR preference in line drawing, in contrast to Lieblich et al.’s (1975) study, and again, this difference could be due to the online setup of the questionnaire.

Our initial expectation was that preferences in Turkish culture would be closer to Iranian rather than German culture, but this did not seem to be the case in the present study. The total scores were negative (i.e., indicating LR direction) for both German and Turkish participants and close to zero (i.e., indicating no preference) for Iranian participants. More specifically, the preferences in object naming, horizontal timeline, and action-moving items were similar to those in Germany. On the other hand, Turkish and Iranian preferences were closer to each other compared to German preferences in bicycle drawing and line drawing. This pattern suggests that certain cultural directional preferences in Turkish culture more tightly linked to the reading direction (i.e., habits that require scanning of horizontally aligned objects/events) tend more to LR. Other preferences that are less influenced by reading direction and more ingrained in the culture may tend towards more RL patterns, because the LR reading direction has only been relatively recently established in Turkey, i.e., for about four generations.

As a result, the CDPQ successfully captured differences in cultural directional preferences across samples in an online setup. This suggests the relative effectiveness of this approach, namely, the pseudo-experimental manipulation of testing of three different cultures, with one being predominantly LR in cultural directional preferences, one being predominantly RL, and the third being mixed between reading direction and cultural directional preferences. Based on these results, the next question was whether such cultural differences would affect SNAs.

### Directional SNAs are influenced by cultural directional preferences

The differences between German and Turkish culture in the SNARC effect, despite the common LR reading direction, strongly suggest that factors other than reading direction are potentially involved in the emergence of SNAs. Turkish CDPQ scores are less LR than German ones, suggesting that these factors could be cultural directional preferences. The influence of cultural directional preferences was previously proposed in several studies (see Nuerk et al., 2015; Patro et al., 2016a, 2016b; Göbel et al., 2018) to explain the emergence of SNAs in preschool children. In the present study, we further suggest that the influence of cultural directional preferences is not merely a transient phase during early preschool development, where reading direction is not (well) developed. Instead, it seems that, even in adults, cultural directional preferences influence SNAs beyond reading direction.

The hypothesized reason for the regular but weaker (compared to German culture) SNARC effect in Turkish culture is that there are RL cultural directional preferences (at least more RL as compared to Germany), even though the reading direction is LR. The underlying reason for the absence of strong and consistent LR directionalities in Turkish culture could be related to the script change around a hundred years ago. However, it could be argued that participants in the present sample were not influenced by this change directly. Note that in 1928 (i.e., just before the time of the Latin alphabet revolution), only 8% of the Turkish population was literate (Yılmaz, 2011), indicating that, even at that time, only a small portion of the population was directly influenced by the change. Importantly though, the reading direction of a culture infiltrates many organizations in the cultural environment and typically covaries with the cultural directionalities. Therefore, (semi-) illiterate individuals can be still influenced by their socio-cultural environment, which naturally involves directionalities (see Nuerk et al., 2015, for a review). Before the Latin alphabet revolution, Turkish culture was probably a more consistent RL culture due to not only the RL Arabic-Farsi alphabet but also the Middle-Eastern and Islamic RL influence on the cultural environment. The literacy rate increased dramatically after the Latin alphabet revolution, eventually influencing the directional preferences in the cultural environment. Nevertheless, some RL habits might have remained, especially those not directly linked to reading direction. Considering that the reading direction is LR, these remaining RL habits might create an inconsistency in associations of space and sequence or space and magnitude (see Patro et al., 2016a, for how such influences may develop), which may be the cause of weaker LR SNAs in Turkish culture.

The differences between Turkish and Iranian culture in the SNARC effect in both tasks suggest that reading direction might also be involved in the emergence of the SNAs. As demonstrated previously in the literature, RL reading habit has a weakening influence on the regular SNARC effect compared to LR reading habit (e.g., Dehaene et al., 1993; Shaki & Fischer, 2008; Lopiccolo & Chang, 2021) and has even been claimed by some to reverse the effect (e.g., Shaki et al., 2009; Zebian, 2005). Nevertheless, when we evaluate the literature overall, the influence of reading habits on the SNARC effect remains unclear due to inconsistent findings among RL readers as well as the absence of evidence of a causal relationship between reading activity and number processing (see Pitt & Casasanto, 2020).

The group-level regular SNARC effect was significant for German and Turkish participants in both PJ and MC tasks. This finding was unsurprising for the German sample since the effect has been demonstrated in LR cultures several times with both tasks (see Wood et al., 2008). In contrast, Bulut et al. (2023) reported no SNARC effect in a Turkish sample with a PJ task¸ however, it should be noted that there are three major differences between Bulut et al. (2023) and the current study. First, in the present study, the number of participants was considerably higher, second, the number of trials per response-to-key assignment was two-fold as in Bulut et al. (2023). These two parameters are highly influential in increasing the sensitivity to detect an existing SNARC effect (Cipora & Wood, 2017). Finally, Bulut et al. (2023) conducted a lab study, while the present study was run online. However, it is unlikely that the difference in the results would originate from online or lab administration because the SNARC was successfully replicated in an online setup previously (e.g., Cipora et al., 2019a). Future investigations need to explore the reason for the difference between the results, but at present, lower power and lower measurement precision seem likely candidates for previous null effects. Also, one should consider that the Turkish SNARC was considerably smaller in size compared to the German one, therefore, the reason for previous null effect findings in the Turkish sample (Bulut et al., 2023) is likely a conjunction of lower power and the effect being smaller (consequently, even more difficult to detect with low power).

In Iranian culture, the regular group-level SNARC effect was significant in PJ and, even though, descriptively, being regular (i.e., negative slope), it was not significant in the MC task. Note that Bayesian statistics provided inconclusive evidence for both tasks in the Iranian group-level SNARC effect. Previous studies also demonstrated conflicting evidence for the SNARC effect at the group level in RL cultures; some reported a reverse SNARC effect (Shaki et al., 2009, in Palestinian participants; Zebian, 2005, in Lebanese participants), others reported null effects (Shaki et al., 2009, in Israeli participants; Dehaene et al., 1993, Exp. 7, in Iranian participants; Lopiccolo & Chang, 2021, in Jordanian participants), and yet others reported a regular SNARC effect (Hochman et al., 2024; Zohar-Shai et al., 2017 in Israeli participants; Feldman et al., 2019 in Israeli children; Supplementary Material of Cipora et al., 2019a, in Iranian participants; Shaki & Gevers, 2011, in Israeli participants). Considering these and the present study’s findings, it seems that more recent studies report regular SNARC effects more frequently, even in RL cultures, than earlier ones. How could a regular SNARC effect be explained in an RL culture? As we discussed above in cultural directionalities, a possible explanation is that LR directionality is innate and overwritten by culture (Adachi, 2014; Drucker & Brannon, 2014; McCrink & de Hevia, 2018; Rugani et al., 2015). Alternatively, instead of overwriting (by deleting the innate biases), cultural directionalities may diversify SNAs. This suggests that innate biases on SNAs are still present in adult life (see *The Brain’s Asymmetric Frequency Tuning* hypothesis in Felisatti et al., 2020) and coexist with culture-dependent representations.

Even though culture clearly influences individuals’ responses, this influence might not be strong enough to reverse the SNARC effect, but only weaken it (i.e., null or weak regular effects). Furthermore, global culture is dominated by LR cultures, as discussed above. With increasing globalization, populations from both LR and RL cultures are exposed to globalized cultural directional preferences. Support for this notion is that more recent studies (i.e., from a more globalized world) found a weakening of the effect, rather than a complete reversal. Moreover, some studies demonstrated the influence of experience with LR culture on the SNARC effect. For instance, Deheane et al. (1993) demonstrated a significant relationship between the time spent in France and the SNARC effect of Iranian participants; the more time in France, the stronger the regular SNARC effect. Furthermore, the level of SNARC effect was shown to be related to participants’ status as either monoliterate (in RL languages) or biliterate (in both LR and RL languages) (see Zebian, 2005; Lopiccolo & Chang, 2021). In the present study, more than half of the Iranian participants reported speaking English as a foreign language and most stated they were somewhat familiar with LR cultures. Although these findings were in line with the globalization explanation of the absence of reverse SNARC effects in RL cultures, the familiarity of the Iranian participants with LR cultures and LR languages overshadows the influence of the cultural directionalities. Therefore, future studies should focus on SNAs among monolinguals/participants who have no/limited experience with LR cultures in general.

Finally, we should also note that Eastern Arabic numbers are written/read in LR fashion, and therefore, during a number-related task, LR activation of the number system might interfere with the responses. The above-mentioned factors may result in inconsistent directionalities in RL cultures, depending on the sample’s characteristics, which in turn resulted in weakened, but not reversed, SNARC effect. A weak SNARC effect could be more susceptible to the power of the study, precision of measurement, and the task at hand, and consequently might produce inconsistent findings, which could explain the divergence of the findings of the SNARC effect in RL cultures. In sum, both the innateness of LR directionality and also, at a more individual level, experience with a predominantly LR global culture may explain why the SNARC effect is merely weakened, not reversed in an RL culture.

### Markedness influencing response codes

Previously, effects of markedness on spatial association with response codes have frequently been reported. One such was the association of parity with response code (even-right and odd-left), which was first reported in German adults (Huber et al., 2015; Nuerk et al., 2004). Morphologically, German includes linguistic markedness in terms of even (“gerade”) being the standard version of the word, and odd (“ungerade”) being the opposite as marked by the prefix (“un”). This corresponds to formal markedness (see Nuerk et al., 2004) and is a strong determinant of the markedness grade. Therefore, we would expect a strong MARC effect in German culture. In the present study, there was some marginally significant (*p* = 0.06) standardized MARC effect, which may hint towards small markedness influence in the German sample. Importantly, Nuerk et al., (2004; see also Iversen et al., 2004, 2006) showed that the MARC effect is more consistent when the stimuli are presented in verbal notation, rather than in the Arabic notation used in this study.

There was no significant MARC effect in Turkish (see also Bulut et al., 2023) or Iranian samples. The morphological structure of markedness is lacking in Turkish (“tek”—odd, “çift”—even) and Farsi (“fard”—odd, “zoj”—even), which could be an explanation for the absence of MARC in these cultures. At the same time, the morphological structure of the language for parity has not always been found to affect the MARC effect (Cipora et al., 2019a, Supplementary Material).

Overall, the MARC effect with Arabic digits was not always replicated, as the effect is small in size and shows interindividual variability (see Huber et al., 2015). The current study adds to these inconsistent results.

### Inter- and intraindividual prevalence of the SNARC effect across cultures

Intraindividual prevalence of the cognitive effects is typically overlooked. To investigate the reliability of an individual SNARC slope, Cipora et al., (2019b) suggested the H0 bootstrapping approach (see also Roth et al., 2024b). Using this method, we found that the reliable SNARC effect was observed most frequently in German (57% in PJ and MC), less in Turkish (38% in PJ and 46% in MC), and the least in Iranian (29% in PJ and 39% in MC). The proportion of reliable SNARC effect was found to be higher in German culture compared to the previously reported values from LR cultures (e.g., ≤ 45% in Cipora et al., 2019b; 42% in Hohol et al., 2022 for PJ, 42% in Hohol et al., 2020 Supplementary Material for MC). Although the reason for this higher proportion is not clear, it could be that participants performed both PJ and MC (see Supplementary Material Tables 15 and 16), whereas, in previous studies, participants only performed the PJ. Overall, these findings indicated that cultural directionalities, directional SNAs (i.e., the SNARC effect), and the prevalence of participants revealing a reliable SNARC effect systematically differed between cultures.

One important point we should address is that although the SNARC and CDPQ findings were comparable regarding the cultural differences, the CDPQ total scores did not correlate with individual SNARC slopes. This finding is not surprising considering that Roth et al. (2024b) demonstrated that a SNARC effect was present at the group level in their study, but was not stable over time for individual participants. Therefore, the absence of correlation between CDPQ and SNARC slopes is not necessarily a result of CDPQ’s failure to predict the SNARC effect, but rather the poor stability of the individual SNARC slopes. A correlation between two measures in which one fluctuates unsystematically will be weak at best. In line with Roth et al. (2024b), we successfully captured the cross-cultural differences at the group level in both CDPQ and the SNARC, while correlations based on individual data did not show the same pattern. Note that further research is needed to fully establish reliability and validity of the CDPQ therefore conclusions should be made with caution. Nevertheless, the findings are promising and suggest that CDPQ could be a new tool to assess the cross-cultural differences in horizontal directional preferences.

### Differences between PJ and MC

Examining the SNARC effect with PJ and MC allowed us to generalize the influence of cultural directionalities on both tasks. Comparing the slopes of these tasks and examining their relationship in a within-design is important in understanding whether they measure the same underlying construct. In the present study, there was no difference between the PJ and MC slopes in any of the cultures, and neither was any correlation observed. The correlation was similarly not significant between the PJ and MC slopes in the majority of the previous studies (see Cipora, 2014; Didino et al., 2019; Fattorini et al., 2015; Georges et al., 2017). Only two studies reported a significant correlation (Cheung et al., 2015; Cipora, 2014, only in an unimanual setup). It seems that even if there is a correlation between the two slopes, it is probably relatively weak, and requires a large sample to detect (e.g., Cheung et al., 2015 with 125 participants), which prompts a more fundamental question regarding the construct validity of SNA as an individual latent characteristic emerging across different tasks.

### Finger counting habits cannot explain the sample differences in SNAs

Finger-counting habits are considered to influence the SNARC effect (Fischer, 2008; Fischer & Brugger, 2011). Although there is no conclusive evidence for this hypothesis (e.g., Prete & Tommasi, 2020; Cipora et al., 2019a, Supplementary Material; Hohol et al., 2022), we identified individuals’ finger-counting direction to examine its influence on the SNARC effect, since such habits show variance across cultures (see Lindemann et al., 2011).

We were unable to replicate Fischer’s (2008) findings in any of the cultures. In line with other studies (Fabbri, 2013; Hohol et al., 2022; Prete & Tommasi, 2020), the SNARC slope of left- and right-starters did not differ significantly. Bayesian analysis supported this lack of differences between the two groups. Although most participants reported stable finger-counting habits (88% in German, 77% in Turkish, and 95% in Iranian), no link was found between the stability of finger-counting habits and the level of the SNARC effect, similar to Hohol et al., (2020; 75% in Polish participants). It is important to note that in the present study, finger-counting habits were collected via self-report in an online setup, an approach that was shown to be inadequate in some studies (Lucidi & Thevenot, 2014; Morrissey & Hallett, 2018; Wasner et al., 2014). Therefore, more controlled lab studies in which participants physically count their fingers repeatedly are needed to accurately capture these habits and their stability for studies focusing on the relationship between finger-counting and the SNAs.

## Limitations

We acknowledge some limitations of the study due to its online nature. Although the sample sizes were very similar in each groups at the beginning of the study, there was more data loss in Iran compared to other countries (e.g., some of the participants’ data were not entirely stored, etc.). Although we believe that this issue should not be a problem with the current study’s sample size (e.g., the minimum detectable effect size would be e.g., 0.41 in German-Iranian comparison for 0.80 power (alpha = 0.05, ratio = 130/75, two sample, two- sided, Welch test), findings should be interpreted with caution since the data loss was unequal across groups.

One should also note that the RTs may not be precise in online studies due to different browsers (Bridges et al., 2020) which might influence the measurement precision of the SNARC effect in the current study. At the same time, the SNARC effect has been successfully replicated in many online studies (e.g., Cipora et al., 2019a; Roth et al., 2024c, 2024d). We recorded the operating systems of the participants and no systematic difference was observed across samples (e.g., more than 75% of participants were using Windows 10 in all samples).

## Conclusion

The present study supported our cultural directionality hypothesis, which stated that, in addition to reading direction, cultural directional preferences influence SNAs. Cultural directional preferences have previously been suggested to play a role in the emergence of SNAs in preliterate children, however, we provide evidence for the first time that such preferences influence SNAs in adults. The cultural environment’s effect on cognition is highly complex and therefore should be examined from a comprehensive perspective. We suggest that examining cultural directionalities as a whole rather than focusing only on reading direction can help us understand the influence of culture on SNAs.

## Supplementary Information

Below is the link to the electronic supplementary material.

Supplementary file1 (PDF 231 KB)

## Data Availability

The data, analyses, and preregistration are available in The Open Science Framework (OSF) at https://osf.io/dk4we/.

## References

[CR1] Adachi, I. (2014). Spontaneous spatial mapping of learned sequence in chimpanzees: Evidence for a SNARC-like effect. *PLoS ONE,**9*(3), e90373. 10.1371/journal.pone.0090373 24643044 10.1371/journal.pone.0090373 PMC3958337

[CR02] Bae, G. Y., Choi, J. M., Cho, Y. S., & Proctor, R. W. (2009). Transfer of magnitude and spatial mappings to the SNARC effect for parity judgments. *Journal of Experimental Psychology: Learning Memory, and Cognition, 35*(6), 1506–1521. 10.1037/a0017257 10.1037/a0017257 19857020

[CR2] Bridges, D., Pitiot, A., MacAskill, M. R., & Peirce, J. W. (2020). The timing mega-study: Comparing a range of experiment generators, both lab-based and online. *PeerJ,**8*, e9414. 10.7717/peerj.9414 33005482 10.7717/peerj.9414 PMC7512138

[CR3] Bulf, H., de Hevia, M. D., & Macchi Cassia, V. (2016). Small on the left, large on the right: Numbers orient visual attention onto space in preverbal infants. *Developmental Science,**19*(3), 394–401. 26074348 10.1111/desc.12315

[CR4] Bulut, M., Hepdarcan, I., Palaz, E., Cetinkaya, H., & Dural, S. (2023). No SNARC effect among left- to-right readers: Evidence from a Turkish sample. *Advances in Cognitive Psychology,**19*(3), 224–236. 10.5709/acp-0394-x

[CR5] Cheung C.-N., Ayzenberg V., Diamond R. F. L., Yousif S. R., Lourenco S. F. (2015). Probing the mental number line: A between-task analysis of spatial-numerical associations. In: Noelle D. C., Dale R., Warlaumont A. S., Yoshimi J., Matlock T., Jennings C. D. (Eds.), *Proceedings of the 37th Annual Conference of the Cognitive Science Society* (pp. 357–362). Lawrence Erlbaum.

[CR6] Chokron, S., & De Agostini, M. (1995). Reading habits and line bisection: A developmental approach. *Cognitive Brain Research,**3*(1), 51–58. 10.1016/0926-6410(95)00018-6 8719022 10.1016/0926-6410(95)00018-6

[CR7] Cipora, K. (2014). Between-task consistency of the SNARC effect. XXXIInd European Workshop on Cognitive Neuropsychology, Bressanone, Italy.

[CR8] Cipora, K., van Dijck, J.‑P., Georges, C., Masson, N., Goebel, S. M., Willmes, K., Pesenti, M., Schiltz, C., & Nuerk, H.‑C. (2019b). *A minority pulls the sample mean: on the individual prevalence of robust group-level cognitive phenomena – the instance of the SNARC effect.* Open Science Framework [Preprint]. 10.31234/osf.io/bwyr3

[CR9] Cipora, K., He, Y., & Nuerk, H.-C. (2020). The spatial–numerical association of response codes effect and math skills: Why related? *Annals of the New York Academy of Sciences,**1477*(1), 5–19. 10.1111/nyas.14355 32348577 10.1111/nyas.14355

[CR10] Cipora, K., & Nuerk, H.-C. (2013). Is the SNARC effect related to the level of mathematics? No systematic relationship observed despite more power, more repetitions, and more direct assessment of arithmetic skill. *The Quarterly Journal of Experimental Psychology,**66*(10), 1974–1991. 10.1080/17470218.2013.772215 23473520 10.1080/17470218.2013.772215

[CR11] Cipora, K., Patro, K., & Nuerk, H.-C. (2018). Situated influences on spatial–numerical associations. In T. Hubbard (Ed.), *Spatial Biases in Perception and Cognition* (pp. 41–49). Cambridge University Press.

[CR12] Cipora, K., Soltanlou, M., Reips, U.-D., & Nuerk, H.-C. (2019a). The SNARC and MARC effects measured online: Large-scale assessment methods in flexible cognitive effects. *Behavior Research Methods,**51*(4), 1676–1692. 10.3758/s13428-019-01213-5 30805864 10.3758/s13428-019-01213-5

[CR13] Cipora, K., & Wood, G. (2017). Finding the SNARC instead of hunting it: A 20*20 Monte Carlo investigation. *Frontiers in Psychology,**8*, 1194. 10.3389/fpsyg.2017.01194 28769840 10.3389/fpsyg.2017.01194 PMC5513957

[CR14] de Hevia, M. D., Girelli, L., Addabbo, M., & Cassia, V. M. (2014). Human infants’ preference for left-to-right oriented increasing numerical sequences. *PLoS ONE,**9*(5), e96412. 10.1371/journal.pone.0096412 24802083 10.1371/journal.pone.0096412 PMC4011793

[CR15] Dehaene, S., Bossini, S., & Giraux, P. (1993). The mental representation of parity and number magnitude. *Journal of Experimental Psychology: General,**122*(3), 371–396. 10.1037/0096-3445.122.3.371

[CR16] Di Giorgio, E., Lunghi, M., Rugani, R., Regolin, L., Dalla Barba, B., Vallortigara, G., & Simion, F. (2019). A mental number line in human newborns. *Developmental Science,**22*, e12801. 10.1111/desc.12801 30676679 10.1111/desc.12801

[CR90] Didino, D., Breil, C., & Knops, A. (2019). The influence of semantic processing and response latency on the SNARC effect. *Acta Psychologica, 196*, 75–86. 10.1016/j.actpsy.2019.04.008 31004938 10.1016/j.actpsy.2019.04.008

[CR17] Didino, D., Brandtner, M., Glaser, M., & Knops, A.(2023). Probing the dual-route model of the SNARC effect by orthogonalizing processing speed and depth. *Experimental Psychology, 70*(1):1–13. 10.1177/0022022111435098 10.1027/1618-3169/a000577 PMC10388237 37039504

[CR18] Dienes, Z. (2021). How to use and report Bayesian hypothesis tests. *Psychology of Consciousness: Theory, Research, and Practice,**8*(1), 9–26. 10.1037/cns0000258

[CR19] Drucker, C. B., & Brannon, E. M. (2014). Rhesus monkeys (Macaca mulatta) map number onto space. *Cognition,**132*(1), 57–67. 10.1016/j.cognition.2014.03.011 24762923 10.1016/j.cognition.2014.03.011 PMC4031030

[CR20] Ebersbach, M., Luwel, K., & Verschaffel, L. (2014). Further evidence for a spatial-numerical association in children before formal schooling. *Experimental Psychology,**61*(4), 323–329. 10.1027/1618-3169/a000250 24351987 10.1027/1618-3169/a000250

[CR21] Fabbri, M. (2013). Finger counting habits and spatial-numerical association in horizontal and vertical orientations. *Journal of Cognition and Culture,**13*(1–2), 95–110. 10.1163/15685373-12342086

[CR22] Faghihi, N., Garcia, O., & Vaid, J. (2019). Spatial bias in figure placement in representational drawing: Associations with handedness and script directionality. *Laterality,**24*(5), 614–630. 10.1080/1357650X.2018.1561708 30580664 10.1080/1357650X.2018.1561708

[CR23] Faghihi, N., & Vaid, J. (2023). Reading/writing direction as a source of directional bias in spatial cognition: Possible mechanisms and scope. *Psychonomic Bulletin & Review,**30*(3), 843–862. 10.3758/s13423-022-02239-1 36604373 10.3758/s13423-022-02239-1

[CR24] Fattorini, E., Pinto, M., Rotondaro, F., & Doricchi, F. (2015). Perceiving numbers does not cause automatic shifts of spatial attention. *Cortex,**73*, 298–316. 10.1016/j.cortex.2015.09.007 26520681 10.1016/j.cortex.2015.09.007

[CR25] Feldman, A., Oscar-Strom, Y., Tzelgov, J., & Berger, A. (2019). Spatial–numerical association of response code effect as a window to mental representation of magnitude in long-term memory among Hebrew-speaking children. *Journal of Experimental Child Psychology,**181*, 102–109. 10.1016/j.jecp.2019.01.001 30735908 10.1016/j.jecp.2019.01.001

[CR26] Felisatti, A., Laubrock, J., Shaki, S., & Fischer, M. H. (2020). A biological foundation for spatial–numerical associations: The brain’s asymmetric frequency tuning. *Annals of the New York Academy of Sciences,**1477*, 44–53. 10.1111/nyas.14418 32645221 10.1111/nyas.14418

[CR27] Fias, W., Brysbaert, M., Geypens, F., & D’Ydewalle, G. (1996). The importance of magnitude information in numerical processing: Evidence from the SNARC effect. *Mathematical Cognition,**2*(1), 95–110. 10.1080/135467996387552

[CR28] Fischer, M. H. (2008). Finger counting habits modulate spatial-numerical associations. *Cortex,**44*(4), 386–392. 10.1016/j.cortex.2007.08.004 18387569 10.1016/j.cortex.2007.08.004

[CR29] Fischer, M. H., & Brugger, P. (2011). When digits help digits: Spatial, numerical associations point to finger counting as prime example of embodied cognition. *Frontiers in Psychology*. 10.3389/fpsyg.2011.00260 22028696 10.3389/fpsyg.2011.00260 PMC3198540

[CR30] Fischer, M. H., Shaki, S., & Cruise, A. (2009). It takes just one word to quash a SNARC. *Experimental Psychology,**56*(5), 361–366. 10.1027/1618-3169.56.5.361 19447752 10.1027/1618-3169.56.5.361

[CR01] Fitousi, D., Shaki, S., & Algom, D. (2009). The role of parity, physical size, and magnitude in numerical cognition: the SNARC effect revisited. *Attention, perception & psychophysics, 71*(1), 143–155. 10.3758/APP.71.1.143 10.3758/APP.71.1.143 19304604

[CR31] Garaizar, P., & Reips, U.-D. (2019). Best practices: Two web browser-based methods for stimulus presentation in behavioral experiments with high resolution timing requirements. *Behavior Research Methods,**51*, 1441–1453. 10.3758/s13428-018-1126-4 30276629 10.3758/s13428-018-1126-4

[CR32] Georges, C., Hoffmann, D., & Schiltz, C. (2017). Mathematical abilities in elementary school: do they relate to number–space associations? *Journal of Experimental Child Psychology,**161*, 126–147. 10.1016/j.jecp.2017.04.011 28527362 10.1016/j.jecp.2017.04.011

[CR33] Gevers, W., Santens, S., Dhooge, E., Chen, Q., van den Bossche, L., Fias, W., & Verguts, T. (2010). Verbal-spatial and visuospatial coding of number-space interactions. *Journal of Experimental Psychology: General,**139*(1), 180–190. 10.1037/a0017688 20121318 10.1037/a0017688

[CR89] Gevers, W., Verguts, T., Reynvoet, B., Caessens, B., & Fias, W. (2006). Numbers and space: a computational model of the SNARC effect. *Journal of Experimental Psychology Human Perception and Performance, 32*(1):32–44. 10.1037/0096-1523.32.1.32 16478324 10.1037/0096-1523.32.1.32

[CR34] Göbel, S. M., McCrink, K., Fischer, M. H., & Shaki, S. (2018). Observation of directional storybook reading infuences young children’s counting direction. *Journal of Experimental Child Psychology,**166*, 49–66. 10.1016/j.jecp.2017.08.001 28865295 10.1016/j.jecp.2017.08.001 PMC5696009

[CR87] Hung, Y. H., Hung, D. L., Tzeng, O. J. L., & Wu, D. H. (2008). Flexible spatial mapping of different notations of numbers in Chinese readers. Cognition, 106 (3), 1441-1450. 10.1016/j.cognition.2007.04.017 17572403 10.1016/j.cognition.2007.04.017

[CR35] Hochman, S., Havedanloo, R., Heysieattalab, S., & Soltanlou, M. (2024, September 5). How does language modulate the association between number and space? A registered report of a cross-cultural study of the SNARC effect. 10.31234/osf.io/sme3z 10.1037/xge0001653 39621408

[CR36] Hoffmann, D., Hornung, C., Martin, R., & Schiltz, C. (2013). Developing number-space associations: SNARC effects using a color discrimination task in 5-year-olds. *Journal of Experimental Child Psychology,**116*(4), 775–791. 10.1016/j.jecp.2013.07.013 24055929 10.1016/j.jecp.2013.07.013

[CR37] Hohol, M., Willmes, K., Nęcka, E., Brozek, B., Nuerk, H.-C., & Cipora, K. (2020). Professional mathematicians do not differ from others in the symbolic numerical distance and size effects. *Scientific Reports*. 10.1038/s41598-020-68202-z 32661271 10.1038/s41598-020-68202-z PMC7359336

[CR38] Hohol, M., Wołoszyn, K., & Cipora, K. (2022). No fingers, no SNARC? Neither the finger counting starting hand, nor its stability robustly affect the SNARC effect. *Acta Psychologica,**230*, 103765. 10.1016/j.actpsy.2022.103765 36242923 10.1016/j.actpsy.2022.103765

[CR39] Hohol, M., Wołoszyn, K., Nuerk, H.-C., & Cipora, K. (2018). A large-scale survey on finger counting routines, their temporal stability and flexibility in educated adults. *PeerJ,**6*, e5878. 10.7717/peerj.5878 30402357 10.7717/peerj.5878 PMC6215439

[CR40] Huber, S., Klein, E., Graf, M., Nuerk, H.-C., Moeller, K., & Willmes, K. (2015). Embodied markedness of parity? Examining handedness effects on parity judgments. *Psychological Research Psychologische Forschung,**79*, 963–977. 10.1007/s00426-014-0626-9 25394996 10.1007/s00426-014-0626-9

[CR03] Ito, Y., & Hatta, T. (2004). Spatial structure of quantitative representation of numbers: Evidence from the SNARC effect. *Memory & Cognition, 32*(4), 662–673. 10.3758/BF03195857 15478760 10.3758/bf03195857

[CR41] Iversen, W., Nuerk, H.-C., Jager, L., & Willmes, K. (2006). The influence of an external symbol system on number parity representation, or what’s odd about 6? *Psychonomic Bulletin & Review,**13*, 730–736. 10.3758/BF03193988 17201377 10.3758/bf03193988

[CR42] Iversen, W., Nuerk, H.-C., & Willmes, K. (2004). Do signers think differently? The processing of number parity in deaf participants. *Cortex,**40*, 176–178. 10.1016/s0010-9452(08)70940-7 15174461 10.1016/s0010-9452(08)70940-7

[CR43] Kebbe, H., & Vinter, A. (2013). How culture, age, and manual dominance affect directionality in drawing side view objects. *Journal of Cross Cultural Psychology,**44*, 160–172. 10.1177/0022022111435098

[CR44] Lieblich, A., Ninio, A., & Kugelmass, S. (1975). Developmental trends in directionality of drawing in Jewish and Arab Israeli children. *Journal of Cross Cultural Psychology,**6*, 504–511. 10.1177/002202217564013

[CR45] Lindemann, O., Alipour, A., & Fischer, M. H. (2011). Finger counting habits in middle eastern and Western individuals: An online survey. *Journal of Cross-Cultural Psychology,**42*(4), 566–578. 10.1177/0022022111406254

[CR46] Lopiccolo, D., & Chang, C. B. (2021). Cultural factors weaken but do not reverse left-to-right spatial biases in numerosity processing: Data from Arabic and English monoliterates and Arabic-English biliterates. *PLoS ONE,**16*(12), e0261146. 10.1371/journal.pone.0261146 34914756 10.1371/journal.pone.0261146 PMC8675726

[CR47] Lorch, R. F., & Myers, J. L. (1990). Regression analyses of repeated measures data in cognitive research. *Journal of Experimental Psychology,**16*(1), 149–157. 2136750 10.1037//0278-7393.16.1.149

[CR48] Lucidi, A., & Thevenot, C. (2014). Do not count on me to imagine how I act: Behavior contradicts questionnaire responses in the assessment of finger counting habits. *Behavior Research Methods,**46*(4), 1079–1087. 10.3758/s13428-014-0447-1 24515889 10.3758/s13428-014-0447-1

[CR49] Maass, A., & Russo, A. (2003). Directional bias in the mental representation of spatial events: Nature or culture? *Psychological Science,**14*(4), 296–301. 10.1111/1467-9280.14421 12807400 10.1111/1467-9280.14421

[CR50] McCrink, K., & de Hevia, M. D. (2018). From innate spatial biases to enculturated spatial cognition: The case of spatial associations in number and other sequences. *Frontiers in Psychology,**9*, 415. 10.3389/fpsyg.2018.00415 29651264 10.3389/fpsyg.2018.00415 PMC5885251

[CR51] McCrink, K., & Opfer, J. E. (2014). Development of spatial–numerical associations. *Current Directions in Psychological Science,**23*(6), 439–445. 10.1177/0963721414549751 26166955 10.1177/0963721414549751 PMC4497778

[CR52] Morrissey, K., & Hallett, D. (2018). Cardinal and ordinal aspects of finger-counting habits predict different individual differences in embodied numerosity. *Journal of Numerical Cognition,**4*(3), 613–635. 10.5964/jnc.v4i3.138

[CR53] Nuerk, H.-C., Iversen, W., & Willmes, K. (2004). Notational modulation of the SNARC and the MARC (Linguistic Markedness of Response Codes) effect. *The Quarterly Journal of Experimental Psychology Section A,**57*(5), 835–863. 10.1080/02724980343000512 10.1080/02724980343000512 15204120

[CR54] Nuerk, H.-C., Patro, K., Cress, U., Schild, U., Friedrich, C. K., & Gobel, S. M. (2015). How space-number associations may be created in preliterate children: six distinct mechanisms. *Frontiers in Psychology*. 10.3389/fpsyg.2015.00215 25798116 10.3389/fpsyg.2015.00215 PMC4350437

[CR55] Nuerk, H.-C., Wood, G., & Willmes, K. (2005). The universal SNARC effect. *Experimental Psychology,**52*(3), 187–194. 10.1027/1618-3169.52.3.187 16076066 10.1027/1618-3169.52.3.187

[CR59] Patro, K., Nuerk, H.-C., & Cress, U. (2016a). Mental number line in the preliterate brain: The role of early directional experiences. *Child Development Perspectives,**10*(3), 172–177. 10.1111/cdep.12179

[CR56] Patro, K., Fischer, U., Nuerk, H.-C., & Cress, U.(2016b). How to rapidly construct a spatial–numerical representation in preliterate children (at least temporarily). *Developmental Science, 19* (1), 126–144. 10.1111/desc.12296 10.1111/desc.12296 25939433

[CR57] Patro, K., & Haman, M. (2012). The spatial-numerical congruity effect in preschoolers. *Journal of Experimental Child Psychology,**111*, 534–542. 10.1016/j.jecp.2011.09.006 22153910 10.1016/j.jecp.2011.09.006

[CR58] Patro, K., Nuerk, H.-C., & Cress, U. (2015). Does your body count? Embodied influences on the preferred counting direction of preschoolers. *Journal of Cognitive Psychology,**27*(4), 413–425. 10.1080/20445911.2015.1008005

[CR60] Pitt, B., & Casasanto, D. (2020). The correlations in experience principle: How culture shapes concepts of time and number. *Journal of Experimental Psychology: General,**149*(6), 1048–1070. 10.1037/xge0000696 31633369 10.1037/xge0000696

[CR61] Prete, G., & Tommasi, L. (2020). Exploring the interactions among SNARC effect, finger counting direction and embodied cognition. *PeerJ*. 10.7717/peerj.9155 32435547 10.7717/peerj.9155 PMC7227642

[CR62] Proctor, R. W., & Cho, Y. S. (2006). Polarity correspondence: A general principle for performance of speeded binary classification tasks. *Psychological Bulletin,**132*(3), 416–442. 10.1037/0033-2909.132.3.416 16719568 10.1037/0033-2909.132.3.416

[CR63] Rashidi-Ranjbar, N., Goudarzvand, M., Jahangiri, S., Brugger, P., & Loetscher, T. (2014). No horizontal numerical mapping in a culture with mixed reading habits. *Frontiers in Human Neuroscience,**8*, 72. 10.3389/fnhum.2014.00072 24605093 10.3389/fnhum.2014.00072 PMC3932419

[CR64] Reips, U.-D. (2002). Standards for Internet-based experimenting. *Experimental Psychology,**49*, 243–256. 10.1026/1618-3169.49.4.243 12455331 10.1026//1618-3169.49.4.243

[CR65] Reips, U.-D. (2021). Web-based research in psychology: A review. *Zeitschrift Für Psychologie,**229*(4), 198–213.

[CR66] Rinaldi, L., Di Luca, S., Henik, A., & Girelli, L. (2014). Reading direction shifts visuospatial attention: An Interactive Account of attentional biases. *Acta Psycholiga,**151*, 98–105. 10.1016/j.actpsy.2014.05.018 10.1016/j.actpsy.2014.05.018 24968311

[CR67] Roth, L., Caffier, J., Cipora, K., Reips, U.-D., & Nuerk, H.-C. (2024c, in press). True colors SNARCing: Semantic number processing is highly automatic. *Journal of Experimental Psychology: Learning, Memory, and Cognition*. Preprint at: https://osf.io/preprints/psyarxiv/aeyn8 10.1037/xlm0001431 39977684

[CR68] Roth, L., Caffier, J., Reips, U.-D., Nuerk, H.-C., Overlander, A. T., & Cipora, K. (2024d, in press). One and only SNARC? Spatial-Numerical Associations are not fully flexible and depend on both relative and absolute number magnitude. *Royal Society Open Science. *Preprint at: https://osf.io/79zsy/10.1098/rsos.241585 PMC11709453 39780972

[CR69] Roth, L., Cipora, K., Overlander, A. T., Nuerk, H.-C., & Ulf-Dietrich, R. (2024a). *Shape of SNARC: How task-dependent are Spatial-Numerical Associations? A highly powered online experiment* [preprint of Stage-1 Registered Report, submitted]. https://osf.io/4wpv6/

[CR70] Roth, L., Jordan, V., Schwarz, S., Willmes, K., Nuerk, H.-C., van Dijck, J.P., & Cipora, K. (2024b). Don’t SNARC me now! Intraindividual variability of cognitive phenomena – insights from the Ironman paradigm. *Cognition 248,* 105781. 10.31234/osf.io/g4bjq 10.1016/j.cognition.2024.105781 38663115

[CR71] Rugani, R., Vallortigara, G., Priftis, K., Regolin, L. (2015). Animal cognition. Number-space mapping in the newborn chick resembles humans' mental number line. *Science 30*;347(6221), 534–536. 10.1126/science.aaa1379 10.1126/science.aaa1379 25635096

[CR72] Schild, U., Steil, J. N., Ulrich, R., & Friedrich, C. (2022). *Children anticipate past events to the left and future events to the right: Evidence from eye movements and time-space compatibility effects.*10.31234/osf.io/cjzd4

[CR73] Schroeder, P. A., Nuerk, H.-C., & Plewnia, C. (2017). Switching between multiple codes of SNARC-like associations: Two conceptual replication attempts with anodal tDCS in sham-controlled cross-over design. *Frontiers in Neuroscience,**11*, 654. 10.3389/fnins.2017.00654 29217996 10.3389/fnins.2017.00654 PMC5703834

[CR74] Shaki, S., & Fischer, M. H. (2024). How do numbers shift spatial attention? Both processing depth and counting habits matter. *Journal of Experimental Psychology. General,**153*(1), 171–183. 10.1037/xge0001493 37796576 10.1037/xge0001493

[CR75] Shaki, S., Fischer, M., & Göbel, S. (2012). Direction counts: A comparative study of spatially directional counting biases in cultures with different reading directions. *Journal of Experimental Child Psychology,**112*(2), 275–281. 10.1016/j.jecp.2011.12.005 22341408 10.1016/j.jecp.2011.12.005

[CR88] Shaki, S., & Fischer, M. H. (2008). Reading space into numbers – a cross-linguistic comparison of the SNARC effect. *Cognition, 108*(2), 590–599 18514179 10.1016/j.cognition.2008.04.001

[CR76] Shaki, S., Fischer, M., & Petrusic, W. (2009). Reading habits for both words and numbers contribute to the SNARC effect. *Psychonomic Bulletin & Review,**16*(2), 328–331. 10.3758/PBR.16.2.328 19293102 10.3758/PBR.16.2.328

[CR77] Shaki, S., & Gevers, W. (2011). Cultural characteristics dissociate magnitude and ordinal information processing. *Journal of Cross-Cultural Psychology,**42*(4), 639–650. 10.1177/0022022111406100

[CR78] Tversky, B., Kugelmass, S., & Winter, A. (1991). Cross-cultural and developmental trends in graphic productions. *Cognitive Psychology,**23*, 515–557. 10.1016/0010-0285(91)90005-9

[CR79] Vaid, J. (1995). Script directionality affects nonlinguistic performance: Evidence from Hindi and Urdu. In I. Taylor & D. R. Olson (Eds.), *Scripts and Literacy* (pp. 295–310). Springer.

[CR80] van Dijck, J. P., & Fias, W. (2011). A working memory account for spatial–numerical associations. *Cognition,**119*, 114–119. 10.1016/j.cognition.2010.12.013 21262509 10.1016/j.cognition.2010.12.013

[CR81] van Dijck, J. P., Fias, W., & Cipora, K. (2022). Spatialization in working memory and its relation to math anxiety. *Annals of the New York Academy of Sciences,**1512*(1), 192–202. 10.1111/nyas.14765 35274298 10.1111/nyas.14765

[CR82] Wasner, M., Moeller, K., Fischer, M. H., & Nuerk, H.-C. (2014). Aspects of situated cognition in embodied numerosity: The case of finger counting. *Cognitive Processing,**15*(3), 317–328. 10.1007/s10339-014-0599-z 24435616 10.1007/s10339-014-0599-z

[CR83] Winter, B., Matlock, T., Shaki, S., & Fischer, M. H. (2015). Mental number space in three dimensions. *Neuroscience and Biobehavioral Reviews,**57*, 209–219. 10.1016/j.neubiorev.2015.09.005 26365108 10.1016/j.neubiorev.2015.09.005

[CR84] Wood, G., Willmes, K., Nuerk, H.-C., & Fischer, M. H. (2008). On the cognitive link between space and number: A meta-analysis of the SNARC effect. *Psychology Science Quarterly,**50*(4), 489–525.

[CR85] Yılmaz, H. (2011). Learning to read (again): The social experiences of Turkey’s 1928 Alphabet Reform. *International Journal of Middle East Studies,**43*(4), 677–697. 10.1017/S0020743811000900

[CR86] Zebian, S. (2005). Linkages between number concepts, spatial thinking, and directionality of writing: The SNARC effect and the reverse SNARC effect in English and Arabic monoliterates, biliterates, and illiterate Arabic speakers. *Journal of Cognition and Culture,**5*(1–2), 165–190. 10.1163/1568537054068660

[CR91] Zohar-Shai, B., Tzelgov, J., Karni, A., & Rubinsten, O. (2017). It does exist! A left-to-right spatial–numerical association of response codes (SNARC) effect among native Hebrew speakers. *Journal of Experimental Psychology: Human Perception and Performance,**43*(4), 719–728. 10.1037/xhp0000336 28182477 10.1037/xhp0000336

