# Supplementary material for: One direction? Cultural aspects of the mental number line beyond reading direction
Source: Psychol Res. 2024 Dec 23;89(1):37. doi: 10.1007/s00426-024-02038-4 (PMC11663824; doi:10.1007/s00426-024-02038-4)
Supplement: Supplementary file 1 — Supplementary file1 (PDF 231 KB) [file 426_2024_2038_MOESM1_ESM.pdf]

Supplementary Materials

Table 1

Detailed information about the languages spoken by bilingual individuals. The datasets from bilingual participants whose second language is written in another direction from the first one (left-to-right for Iran and right-to-left for Germany and Turkey) or whose second language belongs to the Middle Eastern (for Germany) or Western culture (for Turkey and Iran) were excluded

The Sample	Second Native Language	Opposite direction	Middle Eastern (for German) or Western (for Turkish and Iranian) culture	Exclusion	*N*
German	Turkish	no	Middle Eastern	**excluded**	**3**
German	Arabic	yes	Middle Eastern	**excluded**	**1**
German	Spanish	no	-	not excluded	2
German	Greek	no	-	not excluded	2
German	Vietnamese	no	-	not excluded	1
German	Croatian	no	-	not excluded	1
German	Polish	no	-	not excluded	2
German	Bulgarian	no	-	not excluded	1
German	Portuguese	no	-	not excluded	1
German	Russian	no	-	not excluded	1
German	Italian	no	-	not excluded	1
Turkish	German	no	Western	**excluded**	**1**
Turkish	Dutch	no	Western	**excluded**	**1**

Turkish	English	no	Western	**excluded**	**1**
Iranian	Turkish	yes	-	**excluded**	**4**
Iranian	English	yes	Western	**excluded**	**3**
Iranian	Kurdish	no	-	not excluded	1
Iranian	Arabic	no	-	not excluded	1
Iranian	Sangsari	no	-	not excluded	1

Table 2

Foreign languages spoken by the participants. None of the datasets of participants were excluded based on foreign language.

The Language	The Direction	The Sample	N
English	LR	German	126
		Turkish	96
		Iranian	48
Spanish	LR	German	41
		Turkish	1
		Iranian	1
German	LR	Turkish	11
		Iranian	4
French	LR	German	52
		Turkish	1
Arabic	RL	German	3
		Turkish	1
		Iranian	23
Italian	LR	German	12
		Turkish	1
Turkish	LR	German	1
		Iranian	2

Russian	LR	German<br>Turkish<br>Iranian	1<br>2<br>1
Japanese	LR[a]	German<br>Turkish	2<br>3
Dutch	LR	German	3
Farsi	RL	German	1
Mandarin Chinese	LR	German	1
Korean	LR	German	2
Romanian	LR	German	1
Greek	LR	German	1
Norwegian	LR	German	2
Hindu/Urdu	LR/RL	German	1

[a]Japanese is also written from top-to-bottom

The impact of foreign language use

Only three participants in the German and one in the Turkish sample reported knowing a foreign language with RL direction. Forty-six Iranian participants reported knowing a foreign language with LR direction. Therefore, there was only considerable data in the Iranian sample to check whether foreign language use that is in the opposite direction of the native language had any impact on the SNARC slopes. Forty-eight participants reported that they speak English as a foreign language. The majority of them (i.e., 40 participants) reported that they were either actively using English (20 participants) or they had used it in the last five years (20 participants). Eight participants reported that they had not used English actively in the last five years. We considered the latter ones as non-users and excluded their data from this analysis. For the

remaining participants, the correlation analyses showed no significant relationship between foreign language use and the SNARC slopes in any of the tasks (PJ: handwriting: r = .27, p = .089, typing: r = -.07, p = .896, spoken: r = .02, p = .883; MC: handwriting: r = .15, p = .370, typing: r = .07, p = .664, spoken: r = .09, p = .599).

Table 3

Familiarity of participants with the Western (for the Turkish and Iranian samples) and Middle Eastern cultures (for the German sample)

Sample	Not familiar (*N*)	A bit familiar (*N*)	Very familiar (*N*)
German	53.85% (70)	44.62% (58)	0.77% (1)
Turkish	7.14% (8)	60.71% (68)	32.14% (36)
Iranian	2.67% (2)	78.67% (59)	12.50% (14)

Note. 1 participant in the German sample did not answer to this question

Task- and block-order influence on the SNARC effect

Independent samples *t*-tests showed that in none of the tasks and none of the samples, the task order had an effect on the unstandardized or standardized SNARC slopes (see Table 4).

Another series of independent sample *t*-tests indicated that there was no significant effect of block order on PJ unstandardized SNARC slopes (see Table 5). Interestingly, in the German sample, there was a significant effect of block order (t(113.97) = 3.05, p = .003, BF_{10} = 12.39, *adj.* p = .017) on MC SNARC slopes. Based on this analysis, participants who performed the incompatible blocks first showed a stronger SNARC effect (M = -49.37) in the MC task compared to participants who performed the compatible blocks first (M = -16.30). Similar findings were obtained for standardized SNARC slopes in the MC task (German: t(128) = 2.87, p

= 0.005, BF_{10} = 7.30, *adj. p* = .029). This difference was not observed in other samples (see Table 5)

Table 4

Two-sided independent-samples t-test findings indicating that there was no task-order influence on the unstandardized and standardized SNARC slopes

Measure	Sample	Task	*t*	*df*	*p*	BF_{10}
SNARC	German	PJ	0.81	125.41	.421	0.25*
		MC	-0.99	102.23	.323	0.30*
	Turkish	PJ	0.69	109.94	.489	0.25*
		MC	-1.14	103.54	.258	0.36
	Iranian	PJ	-1.44	55.23	.156	0.59
		MC	0.43	71.43	.666	0.26*
Standardized SNARC	German	PJ	0.58	125.15	.560	0.22*
		MC	0.24	107.85	.813	0.19*
	Turkish	PJ	-0.44	109.55	.658	0.22*
		MC	-1.83	109.86	.071	0.88
	Iranian	PJ	-1.24	51.71	.220	0.49
		MC	-0.23	45.89	.818	0.25*

* $p < .05$ indicating a significant finding (frequentist), $BF_{10} < 1/3$ indicating a conclusive finding for the null hypothesis, and $BF_{10} > 3$ indicating a conclusive finding for the alternative hypothesis (Bayesian)

Table 5

Two-sided independent-samples t-test findings indicating the block-order influence on the unstandardized and standardized SNARC slopes

Measure	Sample	Task	*t*	*df*	*p*	BF_{10}
SNARC	German	PJ	-0.16	127.93	.872	0.19*****
		MC	3.05	113.97	.017[a]*	12.39*****
	Turkish	PJ	0.28	108.68	.781	0.21*****
		MC	0.96	105.86	.341	0.29*****
	Iranian	PJ	0.62	64.38	.537	0.28*****
		MC	1.00	48.98	.320	0.38
Standardized SNARC	German	PJ	0.53	126.70	.598	0.21*****
		MC	2.87	128	.029 [a]*	7.30*****
	Turkish	PJ	0.19	105.90	.852	0.21*****
		MC	1.25	109.86	.215	.038
	Iranian	PJ	0.41	72.53	.684	0.26*****
		MC	2.46	72.38	.08 [a]	3.09*****

[a] p is corrected with Holm-Bonferroni for 6 tests

***** $p < .05$ indicating a significant finding (frequentist), $BF_{10} < 1/3$ indicating a conclusive finding for the null hypothesis, and $BF_{10} > 3$ indicating a conclusive finding for the alternative hypothesis (Bayesian)

Table 6

Finger counting habits across samples

Sample (*N*)	Left-starters (*N*)	Right-starters (*N*)	No preference (*N*)
German (130)	43.85% (57)	50.00% (65)	6.15% (8)
Turkish (112)	55.36% (62)	35.71% (40)	8.93% (10)
Iranian (75)	26.03% (19)	64.38% (47)	9.59% (7)

Note. Two participants in the Iranian sample did not respond to the finger counting questions.

Table 7

Finger counting habit and its stability among left- and right- starters across samples

Sample	Left-starters			Right-starters		
	Always (*N*)	Usually (*N*)	Not stable (*N*)	Always (*N*)	Usually (*N*)	Not stable (*N*)
German	43.86% (25)	43.86% (25)	12.28% (7)	43.08% (28)	43.08% (28)	13.85% (9)
Turkish	40.32% (25)	37.10% (23)	22.58% (14)	37.50% (15)	50.00% (20)	12.50% (5)
Iranian	26.32% (5)	68.42% (13)	5.26% (1)	40.43% (19)	55.32% (26)	4.25% (2)

Table 8

Two-sided Jonckheere-Terpstra tests findings comparing the unstandardized SNARC slopes based on the finger-counting stability among left- and right-starters

Sample	Task	Starting Hand	T_{JT}	p	BF_{10}
German	PJ	Left	532	.537	0.43
		Right	654	.881	0.36
	MC	Left	498	.854	0.41
		Right	697	.526	0.36
Turkish	PJ	Left	570	.528	0.32*
		Right	276	.327	0.63
	MC	Left	444	.252[a]	2.44
		Right	290	.159	0.75
Iranian	PJ	Left	25	.142	-
		Right	262	.541	0.60
	MC	Left	38	.833	-
		Right	294	.964	0.98

Note 1. BF's include only always and no preference group.
Note 2. There was not enough observation for BF in Iranian left starters.
[a] p is corrected with Holm-Bonferroni correction for 12 tests
***** $p < .05$ indicating a significant finding (frequentist), $BF_{10} < 1/3$ indicating a conclusive finding for the null hypothesis, and $BF_{10} > 3$ indicating a conclusive finding for the alternative hypothesis (Bayesian)

Table 9

The SNARC effect and the MARC effect in PJ task without applying bilingualism and location filter

Measure	Sample	Mean Slope (SD)	*t*	*p*	BF_{10}
SNARC	German	-5.91 (6.67)	-10.22	< .001*	2.60e+15*
	Turkish	-3.50 (8.54)	-4.40	< .001*	639.77*
	Iranian	-1.96 (7.04)	-2.49	.015*	2.19
Standardized SNARC	German	-0.42 (0.45)	-10.65	< .001*	2.91e+16*
	Turkish	-0.21 (0.45)	-4.98	< .001*	5987.48*
	Iranian	-0.12 (0.47)	-2.23	.029*	1.26
MARC	German	-7.53 (68.04)	-1.28	.204	0.22*
	Turkish	0.56 (85.92)	0.07	.944	0.10*
	Iranian	-8.34 (80.21)	-0.93	.355	0.19*
Standardized MARC	German	-0.12 (0.84)	-1.72	.088	0.40
	Turkish	0.01 (0.85)	0.07	.943	0.10*
	Iranian	-0.11 (0.82)	-1.24	.217	0.26*

Note 1. All tests were two-sided and against zero
Note 2. *df* was 132 for the German, 115 for the Turkish, and 79 for the Iranian sample
***** $p < .05$ indicating a significant finding (frequentist), $BF_{10} < 1/3$ indicating a conclusive finding for the null hypothesis, and $BF_{10} > 3$ indicating a conclusive finding for the alternative hypothesis (Bayesian)

Table 10

The SNARC effect in MC task without applying bilingualism and location filter

Measure	Sample	Mean Slope (SD)	*t*	*p*	BF_{10}
SNARC	German	-32.45 (63.30)	-5.91	< .001*	393308.50*
	Turkish	-26.52 (74.98)	-3.79	< .001*	77.72*
	Iranian	-20.90 (102.21)	-1.83	.071	0.60
Standardized SNARC	German	-0.34 (0.61)	-6.34	< .001*	3.50e+16*
	Turkish	-0.22 (0.60)	-3.96	< .001*	2773.61*
	Iranian	-0.14 (0.58)	-2.17	.033	1.26

Note 1. All tests were against zero
Note 2. *df* was 132 for the German, 115 for the Turkish, and 79 for the Iranian sample
* $p < .05$ indicating a significant finding (frequentist), $BF_{10} < 1/3$ indicating a conclusive finding for the null hypothesis, and $BF_{10} > 3$ indicating a conclusive finding for the alternative hypothesis (Bayesian)

Table 11

The comparison of the SNARC effect across samples without applying bilingualism and location filter

Task	JT[a]	*p*
PJ	21027	< .001*
MC	18974	.064

Note. One-sided JT was performed for the alternative hypothesis of German < Turkish < Iranian order for unstandardized SNARC slopes
[a] $BF_{10} = 2.59$ in German-Turkish and $BF_{10} = 0.36$ in Turkish-Iranian comparison in PJ task
[a] $BF_{10} = 0.17$* in German-Turkish and $BF_{10} = 0.17$* in Turkish-Iranian comparison in MC task
* $p < .05$ indicating a significant finding (frequentist), $BF_{10} < 1/3$ indicating a conclusive finding for the null hypothesis, and $BF_{10} > 3$ indicating a conclusive finding for the alternative hypothesis (Bayesian)

Table 12

The comparison of the SNARC effect between left- and right-starters with two-sided independent samples t-test without applying bilingualism and location filter

Task	Sample	t	df	p	BF_{10}
PJ	German	0.66	122.97	.508	0.23*
	Turkish	-0.52	68.75	.605	0.24*
	Iranian	0.03	34.47	.974	0.27*
MC	German	-1.10	117.99	.275	0.33*
	Turkish	0.18	86.37	.858	0.22*
	Iranian	0.74	34.06	.466	0.33*

***** $p < .05$ indicating a significant finding (frequentist), $BF_{10} < 1/3$ indicating a conclusive finding for the null hypothesis, and $BF_{10} > 3$ indicating a conclusive finding for the alternative hypothesis (Bayesian)

Table 13

The comparison of the SNARC effect based on the stability of finger counting habits with two-sided Jonkheere Terpsta test without applying bilingualism and location filter

Sample	Task	Starting Hand	T_{JT}	*p*
German	PJ	Left	532	.514
		Right	713	.877
	MC	Left	498	.855
		Right	754	.542
Turkish	PJ	Left	592	.377
		Right	276	.349
	MC	Left	458	.144[a]
		Right	290	.170
Iranian	PJ	Left	25	.176
		Right	312	.428
	MC	Left	38	.798
		Right	346	.843

[a] p is corrected with Holm-Bonferroni

***** $p < .05$ indicating a significant finding

Table 14

The comparison of SNARC slopes of PJ and MC tasks (calculated with continuous predictor) with two-sided paired-samples t-test without applying bilingualism and location filter

Sample	*t*	*df*	*p*	BF_{10}
German	0.01	132	.992	0.10*
Turkish	-0.87	114	.385	0.15*
Iranian	-1.02	79	.311	0.20*

* $p < .05$ indicating a significant finding (frequentist), $BF_{10} < 1/3$ indicating a conclusive finding for the null hypothesis, and $BF_{10} > 3$ indicating a conclusive finding for the alternative hypothesis (Bayesian)

Table 15

Individual prevalence of the reliable SNARC effect calculated with H0 bootstrapping in PJ based on task order

Task Order	Sample (N)	Reliable SNARC (*N*)	Reliable reverse SNARC (*N*)	Unreliable SNARC (*N*)
	German (58)	25.38% (33)	2.31% (3)	16.92% (22)
MC-PJ	Turkish (59)	18.75% (21)	5.36% (6)	28.57% (32)
	Iranian (27)	12.00% (9)	2.67% (2)	21.33% (16)
	German (72)	31.54% (41)	2.30% (3)	21.53 % (28)
PJ-MC	Turkish (53)	18.75% (21)	6.25% (7)	22.32% (25)
	Iranian (48)	17.33% (13)	9.33% (7)	37.33% (28)

Table 16

Individual prevalence of the reliable SNARC effect calculated with H0 bootstrapping in MC based on task order

Task Order	Sample (N)	Reliable SNARC (*N*)	Reliable reverse SNARC (*N*)	Unreliable SNARC (*N*)
MC-PJ	German (58)	24.62% (32)	7.69% (10)	12.31% (16)
	Turkish (59)	25.89% (29)	6.25% (7)	20.54% (23)
	Iranian (27)	14.67% (11)	6.67% (5)	14.67% (11)
PJ-MC	German (72)	32.31% (42)	8.46% (11)	14.62 % (19)
	Turkish (53)	20.54% (23)	13.39% (15)	13.39% (15)
	Iranian (48)	24.00% (18)	14.67% (11)	25.33% (19)

The block-order influence on the MARC effect in PJ

The influence of the block order on the MARC effect was examined by performing independent samples *t*-tests on the unstandardized MARC slopes across all samples. Results revealed that performing incongruent blocks first (*M* = -25.25 in the German; *M* = -19.26 in the Turkish, and *M* = -24.62 in the Iranian samples) revealed stronger MARC effects compared to performing congruent blocks first (*M* = 7.96 in the German; *M* = 20.32 in the Turkish, and *M* = 16.43 in the Iranian samples) in PJ (German: *t*(127.52) = 2.85, *p* = .005, *adj. p* = .015; Turkish: *t*(93.89) = 2.50, *p* = .014, *adj. p* = .028; Iranian: *t*(63.61) = 2.50, *p* = .015, *adj. p* = .028). Interestingly, the MARC effect observed among participants who performed the incongruent blocks first was significant at the group level in the German sample as indicated by a one-sample *t*-test against zero (*t*(63) = -3.19, *p* =.002, *adj. p.* = .013). Other MARC effects were not

significant (German sample: congruent blocks first: *t*(65) = 0.24, *p* = .814; Turkish sample congruent blocks first: *t*(63) = 2.09, *p* =.041, *adj. p.* = .162, incongruent blocks first: *t*(46) = -1.54, *p* = .130; Iranian sample congruent blocks first: *t*(35) = 1.22, *p* =.229, incongruent blocks first, *t*(38) = -2.62, *p* =.013, *adj. p.* = .063).

Reliability of the SNARC effect

We calculated the split-half reliability of the SNARC effect in each sample by using the odd-even method based on the presentation order of trials (Cipora et al., 2019a). Unstandardized slopes were re-calculated for odd and even series separately for each participant. The correlation analysis was performed on these recalculated scores. The Pearson correlation coefficients were adjusted for test length by using Spearman-Brown correction. For the PJ task, the reliability of the SNARC effect was .49 in German, .61 in Turkish, and .59 in the Iranian samples. These reliabilities were comparable to the ones calculated in online PJ tasks (.40 and .43 in Hohol et al., 2022; Cipora et al., 2019a, respectively) but rather low compared to in-lab SNARC studies with PJ tasks (.82, .70, .75, and .55 in Cipora et al. 2016; Cipora & Nuerk, 2013; Fattarini et al., 2015; Georges et al., 2017, respectively). For the MC tasks, the reliability of the SNARC effect was .90 in the German, .91 in the Turkish, and .93 in the Iranian sample. To the best of our knowledge, there is no previous reliability report of an MC task. Based on the typical psychometric criteria, we can conclude that the reliability of the MC task was quite high across all cultures. Note that the high reliability in MC could also be a result of the categorical predictor being used in SNARC slope calculations (for a similar calculation for MARC effect reliability see Cipora et al., 2019a which also reports reliability for MARC as .91).

The association of good and bad with the horizontal space

Additionally, Fisher's exact test showed that there was a significant association between the sample and the horizontal space of good and bad, $p < .001$. Bonferroni-corrected post-hoc comparisons revealed that the good-right bad-left association was more frequently selected in the Iranian compared to the German ($p < .001$) and Turkish ($p < .001$) samples. Also, the good-right bad-left association was more frequently selected in the Turkish compared to the German sample ($p = .030$).

Table 17

Operating systems used by participants across samples

Sample (*N*)	Windows 10 (*N*)	Mac OS X 10.15.17 (*N*)	Other (*N*)
German (130)	76.92% (100)	18.46% (24)	4.62% (6)
Turkish (112)	82.14% (92)	13.39% (15)	4.46% (5)
Iranian (75)	93.33% (70)	0.00% (0)	6.67% (5)
